# Household Contact Tracing With Intensified Tuberculosis and Human Immunodeficiency Virus Screening in South Africa: A Cluster-Randomized Trial^[Author-notes fn-0001]^

**DOI:** 10.1093/cid/ciab1047

**Published:** 2021-12-24

**Authors:** Neil A Martinson, Limakatso Lebina, Emily L Webb, Andrew Ratsela, Ebrahim Varavia, Anthony Kinghorn, Sanjay G Lala, Jonathan E Golub, Zama Bosch, Kegaugetswe P Motsomi, Peter MacPherson

**Affiliations:** Perinatal HIV Research Unit, University of the Witwatersrand, Johannesburg, South Africa; Center for Tuberculosis Research, Johns Hopkins University, Baltimore, Maryland, USA; Perinatal HIV Research Unit, University of the Witwatersrand, Johannesburg, South Africa; Medical Research Council International Statistics and Epidemiology Group, London School of Hygiene and Tropical Medicine, London, United Kingdom; Department of Internal Medicine, University of Limpopo, Polokwane, South Africa; Perinatal HIV Research Unit, University of the Witwatersrand, Johannesburg, South Africa; Department of Internal Medicine, Klerksdorp Tshepong Hospital Complex, North West Provincial Department of Health, and University of the Witwatersrand, Johannesburg, South Africa; Perinatal HIV Research Unit, University of the Witwatersrand, Johannesburg, South Africa; Perinatal HIV Research Unit, University of the Witwatersrand, Johannesburg, South Africa; Department of Paediatrics and Child Health, University of the Witwatersrand, Johannesburg, South Africa; Center for Tuberculosis Research, Johns Hopkins University, Baltimore, Maryland, USA; Perinatal HIV Research Unit, University of the Witwatersrand, Johannesburg, South Africa; Perinatal HIV Research Unit, University of the Witwatersrand, Johannesburg, South Africa; Department of Clinical Sciences, Liverpool School of Tropical Medicine, Liverpool, United Kingdom; Malawi-Liverpool-Wellcome Trust Clinical Research Programme, Blantyre, Malawi; Clinical Research Department, London School of Hygiene and Tropical Medicine, London, United Kingdom

**Keywords:** tuberculosis, HIV, screening, diagnosis, randomized controlled trials

## Abstract

**Background:**

Household contact tracing for tuberculosis (TB) may facilitate diagnosis and access to TB preventive treatment (TPT). We investigated whether household contact tracing and intensive TB/human immunodeficiency virus (HIV) screening would improve TB-free survival.

**Methods:**

Household contacts of index TB patients in 2 South African provinces were randomized to home tracing and intensive HIV/TB screening or standard of care (SOC; clinic referral letters). The primary outcome was incident TB or death at 15 months. Secondary outcomes included tuberculin skin test (TST) positivity in children ≤14 years and undiagnosed HIV.

**Results:**

From December 2016 through March 2019, 1032 index patients (4459 contacts) and 1030 (4129 contacts) were randomized to the intervention and SOC arms. Of intervention arm contacts, 3.2% (69 of 2166) had prevalent microbiologically confirmed TB. At 15 months, the cumulative incidence of TB or death did not differ between the intensive screening (93 of 3230, 2.9%) and SOC (80 of 2600, 3.1%) arms (hazard ratio, 0.90; 95% confidence interval [CI], .66–1.24). TST positivity was higher in the intensive screening arm (38 of 845, 4.5%) compared with the SOC arm (15 of 800, 1.9%; odds ratio, 2.25; 95% CI, 1.07–4.72). Undiagnosed HIV was similar between arms (41 of 3185, 1.3% vs 32 of 2543, 1.3%; odds ratio, 1.02; 95% CI, .64–1.64).

**Conclusions:**

Household contact tracing with intensive screening and referral did not reduce incident TB or death. Providing referral letters to household contacts of index patients is an alternative strategy to home visits.

**Clinical Trials Registration:**

ISRCTN16006202.

## Teaser

In South Africa, TB household contacts received letters or home tracing with screening. At 15 months, TB incidence or death did not differ between the intensive screening (93 of 3230, 2.9%) and standard of care (80 of 2600, 3.1%) arms.

Contact tracing of people with tuberculosis (TB) has been advocated as part of TB control for many years [1–3] because it facilitates early diagnosis and treatment of infectious individuals and identifies those who could benefit from TB preventive treatment (TPT) [4]. Although World Health Organization (WHO) and numerous national guidelines recommend household TB contact tracing, these have not been widely implemented in countries with a high TB burden because of limited effectiveness data [5, 6]. The coronavirus disease 2019 (COVID-19) pandemic has severely impacted TB care and prevention programs in sub-Saharan Africa [7] and may have reversed recent improvements in TB diagnosis and treatment [8, 9].

Previous randomized trials have investigated the effectiveness of TB household contact tracing interventions on screening completion, community TB prevalence, and TB notification, showing mixed results [10–13]. Evidence suggests that more intensive TB screening approaches increase diagnostic yield and could reduce transmission by identifying and treating people with subclinical infectious TB earlier [5, 14, 15]. Moreover, universal human immunodeficiency virus (HIV) testing with immediate initiation of antiretroviral therapy (ART), together with TPT, reduces morbidity, mortality, and incident TB disease among people living with HIV [16–18].

We hypothesized that household contact tracing with intensive screening for TB and HIV with supported linkage to treatment and home initiation of TPT could result in earlier TB and HIV diagnosis and treatment and could reduce TB transmission.

## METHODS

### Study Design and Participants

We conducted an open, 2-arm, cluster-randomized trial of household contact tracing and intensive TB/HIV screening in South Africa (ISRCTN16006202). Methods have been described previously (Supplementary Materials, Protocol) [19]. The Mangaung Municipality in Free State Province is predominantly urban with an estimated population of 780 755, an antenatal HIV prevalence of 31.7%, and estimated annual TB incidence in 2019 of 476/100 000. In 2018, the more rural Capricorn Health District in Limpopo Province had an estimated population of 1 338 763 [20], an antenatal HIV seroprevalence in 2015 of 21.6%, and estimated annual TB incidence in 2019 of 201/100 000 [21, 22]. During the study period, there were few programmatic attempts made to identify and screen household contacts for TB.

Study teams identified consecutive eligible index TB patients at government clinics and hospitals within study site boundaries. We included TB patients of any age but required those aged ≥7 years to have laboratory-confirmed pulmonary TB, whereas those aged <7 years could have physician-diagnosed TB of any organ, with or without laboratory confirmation. We additionally included TB patients who died within 8 weeks of TB diagnosis. We excluded institutionalized TB patients and withdrew participants whose households we could not locate or from where no household member could be recruited. A list of household contacts was obtained at enrollment. Households of index patients were defined as people living together within a set of rooms under a contiguous roof linked by doorways or windows through which air moved and where household members had shared airspace by either sleeping overnight at least once or had shared at least 2 meals in the same household as the index case in the 14 days prior to the index case’s diagnosis of TB.

### Randomization, Allocation, and Blinding

Index cases and their households were block-randomized to either intervention or standard of care (SOC) in a 1:1 ratio, stratified by district. Investigator blinding was maintained until after the final participant household follow-up was completed.

### Procedures

In the intervention group, research fieldworkers visited households within 14 days of index TB patient enrollment (maximum 3 attempts), obtaining written individual or parental consent for adults and children aged <18 years, respectively, with assent from older children. A questionnaire was administered to each household member (Supplementary Materials, Questionnaires), and sputum specimens were obtained where possible (but not required from children aged <5 years) and were tested using Xpert and mycobacterial growth indicator tube (MGIT) culture. Household contacts received TST (from a variety of sources due to global shortages), administered and read within 72 hours [23]. Study nurses dispensed the first month of TPT (6 months of daily isoniazid) to participants living with HIV who tested negative for TB, participants not living with HIV with positive TST (≥10 mm), and children aged <5 years. Subsequent TPT was obtained from local clinics.

For household members without a confirmed HIV diagnosis, rapid point-of-care HIV testing was offered to participants aged ≥18 months, and polymerase chain reaction on dried blood spot was provided for children aged <18 months whose maternal HIV status was unknown or positive. Participants living with HIV had a CD4 count measured and were referred to their nearest clinic for assessment and initiation of ART.

Intervention households were visited approximately 3 months after enrollment to support treatment linkage.

In the SOC arm, index TB patients (or their representative, if deceased or a child) were given referral letters for every household member by the recruiting team at the health facility, recommending that each household contact take the letter to their local clinic and be screened for TB and HIV.

### Outcomes

At 15 months after randomization, study teams visited all households, updated the household membership list, and recorded episodes of incident TB and death. We investigated household members for HIV (if untested) and TB (if symptomatic). All children aged ≤14 years had TST placed, read at 48–72 hours.

The primary outcome was time to TB or death, measured among all household members included in the household census at baseline, from 1 month after randomization through the final 15-month ascertainment visit. Primary analysis included all incident TB diagnoses, irrespective of diagnostic method; sensitivity analyses included only bacteriologically confirmed incident cases of TB.

Secondary outcomes were prevalence of TB infection (TST induration ≥10 mm) at month 15 among household children aged ≤14 years, time to initiation of TB treatment, and prevalence of undiagnosed or untreated HIV at month 15. Primary analyses for all outcomes were restricted to household contacts resident at baseline enumeration; supplementary analyses included all household contacts regardless of baseline residency. In protocol-specified subgroup analysis, we compared outcomes by trial site and TST positivity by household contact age (<5 years, ≥5 years).

The University of Witwatersrand Human Research Ethics Committee (Medical) and the London School of Hygiene and Tropical Medicine granted ethical approval.

### Statistical Methods

Assuming a mean household size of 5.5 and a primary outcome incidence of 2000/100 000 person-years, 1200 index cases per site (total 2400) provided 80% power to detect a 30% overall difference in the primary outcome between groups with alpha 0.05 and intracluster correlation coefficient 0.3. All statistical analyses were performed using Stata v16 (StataCorp, College Station, TX). Analyses were done on an intention-to-treat basis.

This study is reported following CONSORT guidelines for cluster-randomized trials (Supplementary Materials, Checklist). We summarized baseline index and household characteristics by trial arm. For the primary outcome, follow-up time began 1 month after randomization (to avoid counting prevalent TB cases) and ended at the month-15 visit or the date of TB or death. Cox proportional hazards regression with robust standard errors was used to assess the impact of the intervention on the primary outcome, with a time-by-treatment interaction term fitted to assess the proportionality assumption. Logistic regression with generalized estimating equations was used to assess the impact of the intervention on binary outcomes. Interaction terms were fitted to assess effect modification in planned subgroup analyses.

## RESULTS

**Figure 1.**
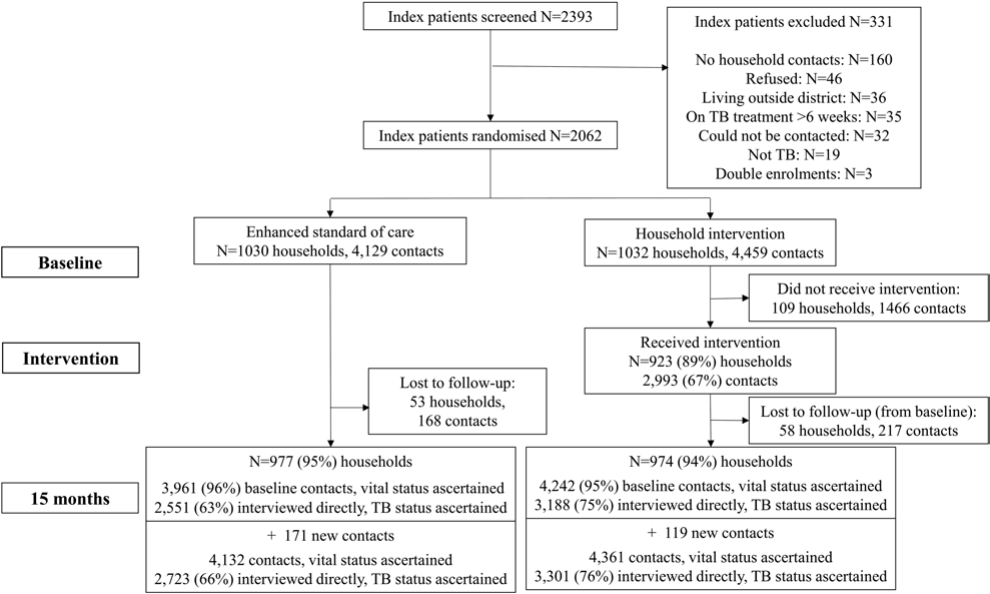
Consort diagram of cluster-randomized trial. Abbreviation: TB, tuberculosis.

From December 2016 through March 2019, we approached 2393 potentially eligible TB index patients, of whom 2062 were randomized (Figure 1). Characteristics of index cases were balanced between arms (Table 1). There were 4459 household contacts identified in the intervention arm and 4192 in the SOC arm (Table 2).

**Table 1.** Characteristics of Index Patients Randomized by Site and Trial Arm

	Study Site		Trial Arm	
Characteristic	Mangaung (N = 1074)	Capricorn (N = 988)	Standard of Care (N = 1030)	Intervention (N = 1032)
Age, median (IQR), years	37 (28–48)	38 (29–48)	37 (28–48)	37 (28–48)
Age group, years				
<8	19 (2%)	26 (3%)	25 (2%)	20 (2%)
8–14	9 (1%)	11 (1%)	14 (1%)	6 (1%)
15–19	55 (5%)	36 (4%)	48 (5%)	43 (4%)
20–29	249 (23%)	179 (18%)	211 (20%)	217 (21%)
30–39	323 (30%)	337 (34%)	325 (32%)	335 (32%)
40–49	191 (18%)	209 (21%)	200 (19%)	200 (19%)
50+	228 (21%)	190 (19%)	207 (20%)	211 (20%)
Sex, male	666 (62%)	573 (58%)	618 (60%)	621 (60%)
Employment				
Currently employed	254 (24%)	113 (11%)	179 (17%)	188 (18%)
Not employed	715 (67%)	679 (69%)	712 (69%)	682 (66%)
Student/child	81 (8%)	93 (9%)	86 (8%)	88 (9%)
Other	24 (2%)	103 (10%)	53 (5%)	74 (7%)
Income type				
Salary	216 (20%)	136 (14%)	162 (16%)	190 (18%)
Wage	70 (7%)	41 (4%)	50 (5%)	61 (6%)
Grant	235 (22%)	215 (22%)	226 (22%)	224 (22%)
No income	553 (51%)	596 (60%)	592 (57%)	557 (54%)
Sputum Xpert				
Positive	1053 (98%)	849 (86%)	952 (92%)	950 (92%)
Negative	3 (0.3%)	14 (1%)	6 (1%)	11 (1%)
Not done	18 (2%)	125 (13%)	72 (7%)	71 (7%)
Sputum smear				
Positive	11 (1%)	295 (30%)	148 (14%)	158 (15%)
Negative	8 (1%)	52 (5%)	32 (3%)	28 (3%)
Not done	1055 (98%)	641 (65%)	850 (83%)	846 (82%)
Sputum culture				
Positive	0 (0%)	59 (6%)	22 (2%)	37 (4%)
Negative	4 (0.4%)	6 (1%)	7 (1%)	3 (0.3%)
Not done	1070 (99.6%)	923 (93%)	1001 (97%)	992 (96%)
Drug resistance, Xpert				
Rifampicin resistance detected	114 (11%)	44 (5%)	75 (8%)	83 (9%)
Rifampicin resistance not detected	906 (89%)	805 (95%)	854 (92%)	857 (91%)
Multidrug-resistant TB				
Resistance to rifampicin and isoniazid	2 (22%)	3 (4%)	1 (3%)	4 (9%)
No resistance to rifampicin and isoniazid	7 (78%)	69 (96%)	36 (97%)	40 (91%)
TB diagnosis				
Microbiologically confirmed	1054 (98%)	939 (95%)	990 (96%)	1003 (97%)
Not microbiologically confirmed	20 (2%)	49 (5%)	40 (4%)	29 (3%)
Human immunodeficiency virus status (self-reported)				
Positive	590 (55%)	521 (53%)	555 (54%)	556 (54%)
Negative	421 (39%)	442 (45%)	434 (42%)	429 (42%)
Unknown	63 (6%)	25 (3%)	41 (4%)	47 (5%)
On antiretroviral therapy				
Yes	345 (58%)	381 (73%)	364 (66%)	362 (65%)
No	245 (42%)	140 (27%)	191 (34%)	194 (35%)
Body mass index, mean (standard deviation), kg/m^2^	19 (5)	20 (5)	19 (5)	20 (5)
Karnofsky score,^a^ median (IQR)	80 (70–90)	80 (70–95)	80 (70–90)	80 (70–90)
Smoking (among those aged ≥15 years)				
Current	254 (24%)	113 (12%)	176 (18%)	191 (19%)
Previous	268 (26%)	216 (23%)	238 (24%)	246 (24%)
Never	524 (50%)	622 (65%)	577 (58%)	569 (57%)
Alcohol use (among those aged ≥15 years)	340 (33%)	223 (24%)	263 (27%)	300 (30%)

Abbreviations: IQR, interquartile range; TB, tuberculosis.

The Karnofsky score is a measure of participant’s ability to undertake activities of daily living and ranges from 0 (dead) to 100 (normal; no complaints; no evidence of disease).

**Table 2.** Characteristics of Baseline Household Contacts of Index Patients by Site and Trial Arm

	Site		Trial Arm	
Characteristic	Mangaung (N = 4202)	Capricorn (N = 4386)	Standard of Care (N = 4129)	Intervention (N = 4459)
Age, median (interquartile range), years	19 (9–38)	19 (9–36)	19 (9–37)	19 (9–37)
Sex, male	1778 (42%)	1913 (44%)	1812 (44%)	1879 (42%)
Relationship to index case				
Spouse	363 (9%)	258 (6%)	288 (7%)	333 (7%)
Child	1129 (27%)	1035 (24%)	1066 (26%)	1098 (25%)
Sibling	682 (16%)	919 (21%)	752 (18%)	849 (19%)
Parent/parent-in-law	439 (10%)	509 (12%)	475 (11%)	473 (11%)
Grandparent	165 (4%)	101 (2%)	140 (3%)	126 (3%)
Grandchild	390 (9%)	357 (8%)	363 (9%)	384 (9%)
Other	1034 (25%)	1207 (28%)	1045 (25%)	1196 (27%)
Joined household, past 15 months	398 (9%)	144 (3%)	279 (7%)	263 (6%)
Has TB symptoms	567 (14%)	406 (9%)	453 (11%)	520 (12%)
If TB symptoms, on treatment	31 (5%)	36 (9%)	40 (9%)	27 (5%)
On antiretroviral therapy				
Yes	354 (8%)	142 (3%)	206 (5%)	290 (7%)
No	3783 (90%)	4194 (96%)	3861 (94%)	4116 (92%)
Don’t know	60 (1%)	42 (1%)	53 (1%)	49 (1%)

Missing values: age (n = 30), entered household in past 15 months (n = 17), TB symptoms (n = 39), antiretroviral therapy (n = 13).

Abbreviation: TB, tuberculosis.

A total of 974 (94%) and 977 (95%) households that were randomized to the intervention and SOC arms, respectively, took part in final outcome assessments, with vital status information available for 4242 (95%) and 3961 (96%) household contacts captured in baseline censuses. In households with outcome assessments, an additional 119 (intervention) and 171 (SOC) individuals moved into the household after the baseline census. Supplementary analysis was therefore based on 4361 household contacts in the intervention arm and 4132 in the SOC arms, of whom 3301 (76%) and 2723 (66%), respectively, were interviewed directly at the 15-month visit and had both TB and HIV outcomes ascertained.

Of households randomized to the intervention arm, 923 of 1032 (89%) received the intervention, 516 (96%) in Mangaung and 407 (82%) in Capricorn. In the 923 households where the intervention was provided, 2993 household contacts consented and then received the intervention (median 3 per household; interquartile range [IQR], 2–4). The prevalence of microbiologically confirmed TB among intervention arm household contacts who provided sputum was 3% (69 of 2166). Overall, 13% (368 of 2752) had positive TST results; 763 initiated TPT.

**Figure 2.**
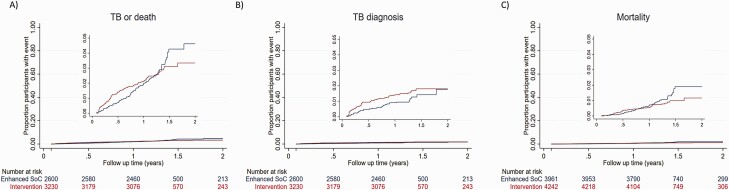
Cumulative hazard of incident TB or death among household contacts of TB patients by trial arm. *A,* Primary trial outcome of incident TB diagnosis or death between month 1 and month 15; inset: *y*-axis truncated to show range of data. *B,* Incident TB diagnosis between month 1 and month 15; inset: *y*-axis truncated to show range of data. *C,* Mortality between month 1 and month 15; inset: *y*-axis truncated to show range of data. Abbreviations: TB, tuberculosis; SOC, standard of care.

The primary outcome, incident TB or death among household contacts present at baseline enumeration, was similar between the household intervention (93 of 3230, 3%) and SOC arms (80 of 2600, 3%; overall incidence 2231/100 000 person-years; hazard ratio [HR], 0.90; 95% confidence interval [CI], .66–1.24; *P* = .54; Table 3). There was some suggestion that the proportional hazards assumption was violated, that is, that the primary outcome incidence was lower in the household intervention arm than in the SOC arm beyond the target follow-up time of 15 months, with cumulative hazard curves crossing at this time point (Figure 2). Allowing the hazard ratio to vary, there was no effect of the intervention either in the first 15 months of follow-up or among those who had their outcome visit beyond 15 months (HR, 1.01; 95% CI, .72–1.42; *P* = .95 and HR, .45; 95% CI, .19–1.05; *P* = .07, respectively).

**Table 3.** Effect of Intervention vs Standard of Care on Trial Outcomes Among Household Contacts Who Were Present at Baseline List of Household Contacts

Outcome	Standard of Care	Household Intervention	
Primary Outcome			Hazard Ratio (95% CI)
Contacts diagnosed with TB	31/2551 (1.2%)	51/3188 (1.6%)	1.33 (.83–2.16)
Contact deaths	49/3961 (1.2%)	42/4242 (1.0%)	0.72 (.47–1.10)
TB or death	80/2600 (3.1%)	93/3230 (2.9%)	0.90 (.66–1.24)
Secondary Outcomes			Odds Ratio (95% CI)
Prevalence of tuberculin skin test positivity (≥10 mm) among children aged ≤14 years	15/800 (1.9%)	38/845 (4.5%)	2.25 (1.07–4.72)
Prevalence of undiagnosed or untreated human immunodeficiency virus infection	32/2543 (1.3%)	41/3185 (1.3%)	1.02 (.64–1.64)

Hazard ratios and odds ratios were calculated with the standard of care arm as the reference group; intracluster correlation coefficients were TB (0.04), death (0.004), TB or death (0.02), tuberculin skin test positivity (0.86), and undiagnosed/untreated human immunodeficiency virus (0.08).

Abbreviations: CI, confidence interval; TB, tuberculosis.

Protocol-specified sensitivity analyses for the primary outcome, including those who had entered the household after the baseline census and based on only bacteriologically confirmed cases of TB, showed similar results (Supplementary Table 4). In protocol-specified subgroup analysis (Supplementary Table 5), there was no difference in the composite primary outcome of incident TB or death at either trial site (Mangaung: HR, 1.26; 95% CI, .81–1.97 and Capricorn: HR, 0.63; 95% CI, .40–1.00), but death was lower among household contacts in Capricorn in the intervention arm compared with the SOC arm (HR, 0.56; 95% CI, .32–0.97).

In the intervention arm, 51 of 3188 (2%) individuals without TB in the baseline census had an episode of incident TB compared with 31 of 2551 (1%) in the SOC arm. Approximately half of the individuals diagnosed with incident TB had biological confirmation (45% in the intervention arm, 52% in the SOC arm), 5 (6%) had multidrug-resistant TB, and 3 (4%) incident TB diagnoses were diagnosed at the final outcome visit (2 in the intervention arm, 1 in the SOC arm). Incidence of TB was 1.24 and 0.92 per 100 person-years among intervention and SOC household contacts, respectively (HR, 1.33; 95% CI, .83–2.16; *P* = .24).

Overall, 69 participants were diagnosed with TB through trial screening: 24 by Xpert alone, 22 by culture alone, 9 by smear alone, and 14 by more than 1 test. Of 69 diagnosed with TB, by the 3 month visit, 37 (54%) were successfully referred for and initiated TB treatment. The median time between date of sample being taken and patient initiating TB treatment was 3 days (IQR, 0–13) and was somewhat higher in the intervention arm compared with the control arm (median, 4 days; IQR, 0–28 vs median, 3 days; IQR, 0–4; *P* = .04).

A total of 91 deaths were ascertained among household contacts in the baseline census population, 42 of 4242 (1%) in the intervention group and 49 of 3961 (1%) in the SOC group. Incidence of mortality was 0.68 and 0.94 per 100 person-years among intervention arm and SOC household contacts, respectively (HR, 0.72; 95% CI, .47–1.10; *P* = .13).

New HIV diagnoses at month 15 occurred with similar frequency in both arms (intervention arm: 11 of 3185, 0.4% vs standard arm: 22 of 2543, 0.9%). At the month-15 visit, 31 participants living with HIV in the intervention arm and 11 participants in the SOC arm were not taking ART. Thus, the prevalence of undiagnosed or untreated HIV at the final visit was comparable between trial arms (both 1%; odds ratio, 1.02; 95% CI, .64–1.64; *P* = .92).

A total of 2271 household contacts seen in person were aged ≤14 years at the time of the final visit. Of these, 1664 (73%) had TST placed (857 in the intervention arm, 807 in the SOC arm); 1645 had the result recorded, with 38 (5%) in the intervention arm and 15 (2%) in the SOC arm testing positive (odds ratio, 2.25; 95% CI, 1.07–4.72; *P* = .03). In protocol-specified subgroup analysis (Supplementary Table 6), TST positivity was higher in the intervention arm compared with the SOC arm among participants aged ≥5 years, but not among participants aged <5 years. At 15 months, of those assessed, 73% of household contacts in the intervention arm reported having taken 6 months of TPT, with 14% taking 1 month or less.

## DISCUSSION

In this trial, a strategy of household contact tracing and intensive screening for TB and HIV did not affect the composite outcome of incident TB or death and was equivalent to providing clinic referral letters to TB index patients. Moreover, we found no difference in prevalence of undiagnosed HIV between arms. A greater proportion of children in the intensive screening arm had latent TB infection by TST testing compared with the SOC arm. Overall household tracing and intensive investigation of household contacts for TB and HIV does not offer sustained benefit beyond the initial screening episode, despite relatively high rates of detection of prevalent undiagnosed HIV and TB.

Our trial differs from previous randomized trials of household contact tracing for TB. In the ZAMSTAR Study, conducted in South Africa and Zambia, household contacts received TB symptom screening, followed by sputum smear microscopy if symptomatic, HIV testing, and TPT [11]. There was weak evidence that household interventions reduced TB prevalence and childhood TB transmission. In Uganda, intervention households received HIV testing and linkage to care, TB symptom screen followed by smear microscopy or Xpert, and with SMS-supported linkage to care [12]. Completion of TB investigation and yield of TB diagnosis were not different between trial arms. In contrast, in a cluster-randomized trial in Vietnam, household contacts of TB patients were invited for clinic-based screening, comprising symptom assessment and chest radiography, followed by smear microscopy and culture if positive [10]. In that trial, there was a 2.5-fold increase in TB treatment registrations in the household contact screening arm compared with the passive case detection arm. In a cluster-randomized trial in Rio de Janeiro, Brazil, supplementing the WHO Directly Observed Therapy, Short Course (DOTS) strategy with more intensive interventions for household contacts (symptom screening, chest X-ray, TST) resulted in reductions in TB case notification rates [13].

Whereas these previous trials limited microbiological investigation to household contacts with a positive symptom screen or abnormal chest X-ray, we evaluated the provision of microbiological testing on household contacts who could provide a sample, prompted by evidence that a substantial fraction of community members with microbiologically confirmed TB have minimal or no symptoms [14, 15]. We successfully obtained sputum samples from 92% of intervention arm adults aged ≥15 years. Compared with previous studies, which mostly used smear microscopy, we used the more sensitive Xpert platform with MGIT culture for sputum testing and made a home visit to intervention arm households to prompt linkage to care [24, 25]. Despite this, a high percentage of household contacts with microbiologically confirmed TB did not initiate TB treatment, emphasizing that new approaches to improving linkage are needed. Our HIV testing strategy intended to identify the anticipated small proportion of people with undiagnosed HIV and link them promptly to ART initiation and TPT, thereby reducing the risk of incident TB disease and death.

Despite achieving high intervention coverage and follow-up, we saw no difference in TB-free survival between arms. There are a number of possible explanations for this. We anticipated that a letter prompting clinic-based screening for household contacts in the SOC arm would be insufficient to achieve satisfactory levels of screening completion and treatment linkage [26]. However, the percentage of household contacts who initiated TB treatment was only slightly higher in the intervention arm (1.6%) compared with the SOC arm (1.2%), perhaps reflecting high motivation for TB screening among household contacts who received letters. It is also possible that in intervention households, TB transmission had already occurred at the time of the intervention. Finally, as the majority of TB transmission is thought to occur outside households [27], high forces of infection within South African communities may overcome the benefits of interventions targeting households. Indeed, in Capricorn, a relatively low annual TB incidence area (for South Africa), there was a suggestion that the intervention was effective in reducing mortality.

Our findings suggest that household contact tracing with home visits and intensive screening for TB and HIV is unlikely to be considered for implementation by national TB programs in low-resourced settings with high TB burden. Although household contact tracing of index TB patients is widely recommended, implementation is often poor due to the substantial resource requirements. Cost studies will be reported separately, but we anticipate resource implications of household visits to be substantial. Conversely, the strategy of providing referral letters for household contacts to take to their local clinics to prompt facility attendance for TB/HIV screening and care is affordable and implementable at scale but requires further implementation research and evaluation.

We found that in the intervention arm, prevalence of latent TB (defined by TST) was 13%, comparable to previous household contact tracing studies from the region [26], and with strong age- and site-specific dependency [28]. At 15 months, TST positivity in children was higher in the intervention arm than in the SOC arm. One possible explanation is differential rates of acceptance of TST between the intervention and SOC arms by site; we did not record data on reasons for refusal of the TST but it may be that those with a strong response previously were reluctant to be retested.

The study had several limitations. The planned sample size was reduced due to budget constraints; follow-up time in 14% of households was reduced in anticipation of South African COVID-19 pandemic-related lockdown (Supplementary Materials, Checklist) [29]. The study was at a time when South African preventive treatment guidelines were in flux, initially requiring a positive TST to initiate TPT and with restrictions on people who could receive TPT. This likely reduced the proportion of individuals not living with HIV aged >5 years who continued TPT beyond the initial study-dispensed month. Not all households allocated to the intervention arm received interventions, mainly due to difficulties locating households. We did not ascertain causes of death. Results may not be generalizable to settings with different healthcare systems.

In conclusion, an intensive household contact tracing and TB/HIV screening intervention did not reduce incident TB or death when compared with a referral letter intervention. TB program managers and policy makers should carefully consider benefits and costs before implementing similar household contact tracing and TB screening interventions. The provision of referral letters to index patients at the time of their TB diagnosis should be the preferred strategy to link household contacts to care in low-resourced settings with high TB burden.

## Supplementary Data

Supplementary materials are available at *Clinical Infectious Diseases* online. Consisting of data provided by the authors to benefit the reader, the posted materials are not copyedited and are the sole responsibility of the authors, so questions or comments should be addressed to the corresponding author.

ciab1047_suppl_Supplementary_Data_S1

ciab1047_suppl_Supplementary_Data_S2

ciab1047_suppl_Supplementary_Data_S3

ciab1047_suppl_Supplementary_Data_S4

ciab1047_suppl_Supplementary_Data_S5

ciab1047_suppl_Supplementary_Data_S6

ciab1047_suppl_Supplementary_Data_S7

## Notes

***Acknowledgments.*** The authors acknowledge the members of the Data and Safety Monitoring Board (DSMB; Derek Sloan, Yunus Moosa, and Locadiah Kuwanda) for their expert oversight and advice.

***Financial support.*** This work was supported by the UK/South Africa Medical Research Council (MRC) Newton Fund (006Newton TB) and Wellcome (200901/Z/16/Z to P. M.). This research was funded in whole, or in part, by the Wellcome Trust [200901/z/16/Z]. For the purpose of Open Access, the author has applied a Creative Commons Attribution License (cc-by) public copyright license to any Author Accepted Manuscript version arising from this submission. A. K. and N. A. M. report trial funding (paid to their institutions) from the South African MRC and UK Government Newton Fund(006Newton TB). N. A. M. reports that the Department of Health, Republic of South Africa, funded all laboratory testing for this trial.
