# Supplementary material for: Household Contact Tracing With Intensified Tuberculosis and Human Immunodeficiency Virus Screening in South Africa: A Cluster-Randomized Trial
Source: Clin Infect Dis. 2021 Dec 24;75(5):849–56. doi: 10.1093/cid/ciab1047 (PMC9477445; doi:10.1093/cid/ciab1047)
Supplement: ciab1047_suppl_Supplementary_Data_S2 [file ciab1047_suppl_supplementary_data_s2.pdf]

Index Case Report Form C

Study ID	________________
Study site:	◯ Mangaung<br>◯ Capricorn
Name of area	________________
C01. Form completed by	◯ Index case<br>◯ Next-of-kin<br>◯ Parent/Legal guardian

Demographics

C02. Date	________________
C03. Site where participant was first recruited or diagnosed	◯ Clinic<br>◯ Hospital out-patient<br>◯ Hospital in-patient<br>◯ TB Laboratory
C04. Date of Admission	________________
C05. Date of Discharge	________________
C06. Deceased?	◯ Yes<br>◯ No
C07. Date of death	________________
C08. Name of clinic or hospital	________________
C09. Age of index case in:	◯ Years<br>◯ Months
C10. __ __ years	________________
C11. __ __ months	________________
C12. Gender of index case	◯ Male<br>◯ Female

C13. Employment status	◯ Currently employed<br>◯ Not employed<br>◯ Student<br>◯ Other
C13.a. Describe Other	__________________________________
C14. Employment industry	◯ Agriculture<br>◯ Mining<br>◯ Construction<br>◯ Manufacturing<br>◯ Transportation<br>◯ Hospitality<br>◯ Unemployed<br>◯ Student<br>◯ Other
C14.a. Describe Other	__________________________________
C15. Index case monthly income type	◯ Salary<br>◯ Wage<br>◯ Grant<br>◯ No income
C16. Index case monthly income amount	◯ R0<br>◯ R1-R999<br>◯ R1000-R4999<br>◯ R5000-R9999<br>◯ R10000+

Basis of diagnosing TB (tick all that apply)

C17. Sputum Xpert	◯ Positive ◯ Negative<br>◯ Not done
C18. Sputum smear	◯ Positive ◯ Negative<br>◯ Not done
C19. Sputum culture	◯ Positive ◯ Negative<br>◯ Not done
C20. Chest X-Ray	◯ Positive ◯ Negative<br>◯ Not done
C21. (Children only)	◯ Clinical diagnosis
C78. LF-LAM	◯ Positive<br>◯ Negative<br>◯ Invalid<br>◯ Not done
C22. How sick was the index case at recruitment?<br><br>(Karnofsky score) __ __%	__________________________________

C23. Weight __ __ __kg

____

C24. Height __ __ __cm

____

C25. Blood pressure __ __ __/__ __ __

____
(Sys/Dia)

C26. MUAC __ __mm

____

C27. Fingerprick HGT __ __.__mmol/L

____
(Changed to 25yrs as of 20 July 2017)

C28. Did you draw blood for HbA1C?
- ◯ Yes
- ◯ No

C29. HbA1C result

____

General Health

C30. Does the index case currently smoke tobacco?
- ◯ Yes (current smoker)
- ◯ No (but smoked previously)
- ◯ Never smoked

C31. Does/did the index case smoke tobacco:
- ◯ Daily
- ◯ Weekly
- ◯ Monthly
- ◯ Every few months
- ◯ Only when drinking alcohol
- ◯ Never smoked

C32. How many cigarettes does/did the index case smoke per day? __ __

____

C33. How old was the index case when he/she started smoking tobacco? __ __

____

C34. Does/did the index case drink alcohol?
- ◯ Yes
- ◯ No

C35. How many units of alcohol does/did the index case drink?

__ __ __ units/week

____
(If NONE record 000. If NOT KNOWN record 999)

C36. Does the index case drink traditional beer?
- ◯ Yes
- ◯ No

C37. HIV sero-status of index case?
- ◯ Positive
- ◯ Negative
- ◯ Unknown

C38. Most recent CD4 count __ __ __ __

(9999 if CD4 count never done)

C39. Date taken

C40. Is/was the index case taking ART for the treatment of HIV?

(If deceased were they taking ART before they died?)

◯ Yes
◯ No

C41. Date started ARV's

C42. Has the index case been referred for ARV initiation?

◯ Yes
◯ No
◯ Already taking ART

C43. Has the index case tested for HIV before?

◯ Yes
◯ No

C44. When was the last HIV test done?

◯ In the last year
◯ Over 1 year ago
◯ Never tested for HIV

Symptom Screen

C45. Cough (days) __ __ __

(If NO record 000. If NOT KNOWN record 999)

C46. Productive cough (days) __ __ __

(If NO record 000. If NOT KNOWN record 999)

C47. Blood stained or bloody cough (days) __ __ __

(If NO record 000. If NOT KNOWN record 999)

C48. Loss of weight (days) __ __ __

(If NO record 000. If NOT KNOWN record 999)

C49. Night sweats (days) __ __ __

(If NO record 000. If NOT KNOWN record 999)

C50. Fever (days) __ __ __

(If NO record 000. If NOT KNOWN record 999)

C51. Has index lived with any person who has had TB in the last 3 years?

◯ Yes
◯ No
◯ Don't know

C52. Did this person with TB at home, take TB treatment?
- ◯ Yes
- ◯ No
- ◯ Don't know

C53. Is this person who had TB deceased?
- ◯ Yes
- ◯ No

Co-Morbid Conditions in the Index Case

C54. Diabetes
- ◯ No Diabetes
- ◯ Diabetes - Insulin injection
- ◯ Diabetes - Oral treatment only
- ◯ Diabetes - Not treated

C55. Hypertension
- ◯ No Hypertension
- ◯ Hypertension - Oral treatment only
- ◯ Hypertension - Not Treated

TB Results
(Only include specimens that were taken two months before to two months after enrolment)

C56. GeneXpert
- ◯ M Tuberculosis detected
- ◯ M Tuberculosis not detected
- ◯ Not done

C56. Date taken (GeneXpert) ______________________________

C57. GeneXpert Rif result
- ◯ Rifampicin resistance detected
- ◯ Rifampicin resistance not detected
- ◯ Not done

C58. Sputum smear result
- ◯ Negative/Not seen
- ◯ Scanty
- ◯ 1+
- ◯ 2+
- ◯ 3+
- ◯ Not done

C58. Date taken (Sputum smear) ______________________________

C59. Sputum culture result
- ◯ Positive for M tuberculosis
- ◯ Negative
- ◯ Contaminated or lab problem
- ◯ Not done

C59. Date taken (Sputum culture) ______________________________

Drug Sensitivity from any result in file or in the lab for this episode of TB

C60. Isoniazid
- ◯ Resistant
- ◯ Sensitive
- ◯ Not done

C61. Rifampicin
- ◯ Resistant
- ◯ Sensitive
- ◯ Not done

C62. If resistant to Isoniazid and Rifampicin, resistant to second-line drugs?
- ◯ Yes
- ◯ No

C63. Resistant to ______________________________

C63. Resistant to ______________________________

C63. Resistant to ______________________________

C64. Other biological specimen
- ◯ Collected
- ◯ Not collected

C65. Specify specimen ______________________________

C66. Date taken (other biological specimen) ______________________________

C67. Result
- ◯ TB (probable/confirmed)
- ◯ No TB
- ◯ Unknown

C68. CXR
- ◯ Done
- ◯ Not done

C69. Result
- ☐ Cavities
- ☐ Bilateral disease

C70. Record anatomical site of TB
- ◯ Pulmonary (lungs/pleura)
- ◯ Extra-pulmonary

C71. Site(s) ______________________________

C72. Has the index case previously been diagnosed with or treated for TB?
- ◯ Yes
- ◯ No

For THIS episode of TB in the Index Case

C73. TB treatment started?

◯ Yes
◯ No

C74. Start date

____

C75. Reason for not starting TB treatment

◯ Deceased
◯ Defaulted
◯ Other

C75. State Other

____

Form Completion

C76. Date form C completed

____

C77. Name of person completing this form

____

Audit Trail

Name of Data Entry Person

____

Date of Data Entry

____
(Kindly change the date every time a change is made to the data)

Form Version: 1.2 dated 22 October 2018

____

Index Census For Household Members Form D

D01. Completed by	◯ Index case<br>◯ Next-of-kin<br>◯ Parent/Legal guardian
D02. Child headed household	◯ Yes<br>◯ No
D03. Total number of household members __ __	______________________
D04 Name	______________________
D05 Participant ID<br><br>(kindly enter the complete ID, inclusive of Study ID)	______________________<br>(x-xx-xxxx-xx format. Changed 19-10-2017)
D06 Age	______________________
D06 Age in:	◯ Years<br>◯ Months<br>◯ UNK
D07 Gender	◯ Male<br>◯ Female
D08 Relationship to index	◯ Husband/Wife/Partner<br>◯ Son/Daughter/Stepchild<br>◯ Brother/Sister<br>◯ Parent (mother/father)<br>◯ Parent-in-law<br>◯ Grandparent<br>◯ Grandchild<br>◯ Other
D08.1. Describe other	______________________
D09 Confirm this person is alive	◯ Yes<br>◯ No
D10 New household member Entered household in past 15 months	◯ Yes<br>◯ No<br>◯ N/A
D10.1. Does this person have TB symptoms now?	◯ Yes<br>◯ No<br>◯ N/A
D11 Cough __ __	______________________

D12 Weight loss __ __ ______________________

D13 Fever __ __ ______________________

D14 Night sweats __ __ ______________________

D15 Is this person taking TB treatment now?
- ◯ Yes
- ◯ No
- ◯ Don't know

D16 Is this person taking ART?
- ◯ Yes
- ◯ No
- ◯ Don't know
- ◯ N/A

D17. To which group was this person randomized to?
- ◯ Household intervention strategy
- ◯ Letter strategy

D18. Randomization number __-__ __-R__ __ __ __-01 ______________________

D19. How many letters were given to household? __ __ ______________________

Form Completion

D20. Date form D completed ______________________

D21. Time completed ______________________

D22. Name of person completing this form ______________________

Some required questions have been skipped!
Kindly review questions
D03, D18 & D19 before proceeding....

Audit Trail

Name of Data Entry Person ______________________

Date of Data Entry ______________________
(Kindly change the date every time a change is made to the data)

Household Census At Baseline Form G

G01. Housing type
- ◯ Brick/Concrete house
- ◯ Apartment
- ◯ Traditional hut/Dwelling
- ◯ Garage
- ◯ 1 room
- ◯ Shack
- ◯ Other

Describe other ______________________

G02. How many dining rooms? ______________________

G03. How many kitchens? ______________________

G04. How many bed rooms? ______________________

G05. How many living rooms? ______________________

G06. How many bathrooms/toilet? ______________________

G07. How many other rooms? [Type 1] ______________________

G08. How many other rooms? [Type 2] ______________________

G09. Total rooms in household ______________________

G10. How many windows are there in the household? ______________________

G11. How many doors are there in the household? ______________________

G12. What are your main sources of energy for cooking? [Primary]
- ☐ Electricity
- ☐ Gas
- ☐ Wood
- ☐ Coal
- ☐ Paraffin
- ☐ Candles
- ☐ Other

G12. What are your main sources of energy for cooking? [Secondary]	☐ Electricity<br>☐ Gas<br>☐ Wood<br>☐ Coal<br>☐ Paraffin<br>☐ Candles<br>☐ Other
G13. What are your main sources of energy for heating? [Primary]	☐ Electricity<br>☐ Gas<br>☐ Wood<br>☐ Coal<br>☐ Paraffin<br>☐ Candles<br>☐ Other
G13. What are your main sources of energy for heating? [Secondary]	☐ Electricity<br>☐ Gas<br>☐ Wood<br>☐ Coal<br>☐ Paraffin<br>☐ Candles<br>☐ Other
G14. What are your main sources of energy for lighting? [Primary]	☐ Electricity<br>☐ Gas<br>☐ Wood<br>☐ Coal<br>☐ Paraffin<br>☐ Candles<br>☐ Other
G14. What are your main sources of energy for lighting? [Secondary]	☐ Electricity<br>☐ Gas<br>☐ Wood<br>☐ Coal<br>☐ Paraffin<br>☐ Candles<br>☐ Other
G15. How many people smoke tobacco inside the household? __ __ __	______________________________<br>(If NONE record 000. If NOT KNOWN record 999)
G16. Access to piped water	◯ Piped water inside the dwelling<br>◯ Piped water within 100m of household<br>◯ Piped water inaccessible<br>◯ Piped water inside the yard<br>◯ Piped water on a community stand < 100m of household
G17. Main source of water for drinking/cooking/cleaning	◯ Government piped water<br>◯ Borehole<br>◯ Spring<br>◯ Dam/pool/stagnant water<br>◯ River or stream<br>◯ Other
Describe other	______________________________

G18. Toilet facilities	◯ Flush toilet<br>◯ Chemical toilet<br>◯ Pit toilet<br>◯ Bucket toilet<br>◯ Other
Describe other	__________________________________

Deceased

G19. Did anyone who lived in this house die in the past year?	◯ Yes<br>◯ No
G20. How many people living in the household died in the last year?	__________________________________

Record the 4 most recent deaths in the household, with the most recent FIRST

G21.1. Age at death [Death 1]	__________________________________
G21.1. Gender [Death 1]	◯ Male<br>◯ Female
G23.1. Died with TB? [Death 1]	◯ Yes<br>◯ No<br>◯ Don't know
G24.1.Died whilst taking TB treatment? [Death 1]	◯ Yes<br>◯ No<br>◯ Don't know
G25.1. Did they ever test HIV-positive? [Death 1]	◯ Yes<br>◯ No<br>◯ Don't know
G26.1. Taking ART at death? [Death 1]	◯ Yes<br>◯ No<br>◯ Don't know

Record the 4 most recent deaths in the household, with the most recent FIRST

G21.2. Age at death [Death 2]	__________________________________
G22.2. Gender [Death 2]	◯ Male<br>◯ Female
G23.2. Died with TB? [Death 2]	◯ Yes<br>◯ No<br>◯ Don't know
G24.2.Died whilst taking TB treatment? [Death 2]	◯ Yes<br>◯ No<br>◯ Don't know

G25.2. Did they ever test HIV-positive? [Death 2]	◯ Yes<br>◯ No<br>◯ Don't know
G26.2. Taking ART at death? [Death 2]	◯ Yes<br>◯ No<br>◯ Don't know

Record the 4 most recent deaths in the household, with the most recent FIRST

G21.3. Age at death [Death 3]	__________________
G22.3. Gender [Death 3]	◯ Male<br>◯ Female
G23.3. Died with TB? [Death 3]	◯ Yes<br>◯ No<br>◯ Don't know
G24.3.Died whilst taking TB treatment? [Death 3]	◯ Yes<br>◯ No<br>◯ Don't know
G25.3. Did they ever test HIV-positive? [Death 3]	◯ Yes<br>◯ No<br>◯ Don't know
G26.3. Taking ART at death? [Death 3]	◯ Yes<br>◯ No<br>◯ Don't know

Record the 4 most recent deaths in the household, with the most recent FIRST

G21.4. Age at death [Death 4]	__________________
G22.4. Gender [Death 4]	◯ Male<br>◯ Female
G23.4. Died with TB? [Death 4]	◯ Yes<br>◯ No<br>◯ Don't know
G24.4.Died whilst taking TB treatment? [Death 4]	◯ Yes<br>◯ No<br>◯ Don't know
G25.4. Did they ever test HIV-positive? [Death 4]	◯ Yes<br>◯ No<br>◯ Don't know
G26.4. Taking ART at death? [Death 4]	◯ Yes<br>◯ No<br>◯ Don't know

Form Completion

G27. Date form G completed

____

G28. Name of person completing this form

____

Some required questions have been skipped!
Kindly review questions
G01 & G09 before proceeding....

Audit Trail

Name of Data Entry Person

____

Date of Data Entry

____
(Kindly change the date every time a change is made to the data)

Baseline Household Member Form H

Participant ID	__________________
Name	__________________<br>(First 2 letters of First name and first 2 letters of Last name)
H01. Has household member consented to be in the study?	◯ Yes<br>◯ No
H02. Date participant enrolled	__________________
Age of household member in:	◯ Years<br>◯ Months
H03. __ __ years	__________________
H04. __ __ months	__________________
Age captured doesn't correlate to that captured in Form D!<br>Kindly review before proceeding...	
H05. Gender of index case	◯ Male<br>◯ Female
H06. Relationship to Index case	◯ Husband/wife/partner<br>◯ Son/daughter/stepchild<br>◯ Brother/sister<br>◯ Parent(mother/father)<br>◯ Parent-in-law<br>◯ Grandparent<br>◯ Grandchild<br>◯ Other
Describe other	__________________
H07. Employment status	◯ Currently employed<br>◯ Not employed<br>◯ Student<br>◯ Other

H08. Employment industry	◯ Agriculture<br>◯ Mining<br>◯ Construction<br>◯ Manufacturing<br>◯ Transportation<br>◯ Hospitality<br>◯ Unemployed<br>◯ Student<br>◯ Other
H09. Household member monthly income type	◯ Salary<br>◯ Wage<br>◯ Grant<br>◯ No income
H10. Household member monthly income amount	◯ R0<br>◯ R1-R999<br>◯ R1000-R4999<br>◯ R5000-R9999<br>◯ R10000+
H11. Over the past 3 months have you shared AIR space with index case?	◯ Yes<br>◯ No
H12. Over the past 3 months how much time did you spend with the index case?	◯ Every now and then<br>◯ Part of the day<br>◯ Most of the day
H13. What activities do you do with the index case?	☐ Meals<br>☐ Watch TV<br>☐ Sleep in the same room<br>☐ Sleep on the same bed<br>☐ Other
Describe other	__________________________________

Quality of Life

H14. In general how do you rate your general health	◯ Very good<br>◯ Good<br>◯ Neither good or bad<br>◯ Poor<br>◯ Very poor
H15. Do you have any difficulties doing the usual activities?	◯ No problems<br>◯ Slight problems<br>◯ Moderate problems<br>◯ Severe problems<br>◯ Unable to do usual activities

General Health

H16. Weight __ __ __.__kg

H17. Height __ __ __cm

H18. Blood pressure __ __ __/__ __ __

Kindly insert leading zero if your reading has only 2 digits.
e.g 120/080, 090/060 etc

H19. MUAC __ __mm

H20. Do you currently smoke tobacco?
- ◯ Yes (currently smoke)
- ◯ No (previously smoked)
- ◯ Never smoked

H21. How often did/do you smoke?
- ◯ Daily
- ◯ Weekly
- ◯ Monthly
- ◯ Every few months
- ◯ Only when drinking alcohol
- ◯ Never smoked

H22. On average, how many cigarettes did/do you smoke per day? __ __ __

(999, if NOT known)

H23. How old were you when you started smoking tobacco? __ __

(99, if NOT known)

H24. Do you drink alcohol now?
- ◯ Yes
- ◯ No

H25. On average, how many units of alcohol; do you drink per week? __ __ __

(999, if NOT known)

H26. Does the household member drink traditional beer?
- ◯ Yes
- ◯ No

H27. What is your HIV status? (before study testing)
- ◯ Positive
- ◯ Negative
- ◯ Not known

H28. Most recent CD4 count __ __ __ __ cells/mm3

(9999, if CD4 count NEVER done)

H29. Date taken

H30. Are you taking ART as treatment for HIV?
◯ Yes
◯ No

H31. Date ART started
____

H32. If ART not started, has the household member been referred for ARV initiation?
◯ Yes
◯ No

H33. Has household member tested for HIV before?
◯ Yes
◯ No

H34. When was your last HIV test done?
◯ In the last year
◯ Over 1 year ago
◯ Never tested for HIV

Symptom Screen

H35. Cough (days) __ __ __
____
(If NO record 000. If NOT KNOWN record 999)

H36. Productive cough (days) __ __ __
____
(If NO record 000. If NOT KNOWN record 999)

H37. Blood stained or bloody cough (days) __ __ __
____
(If NO record 000. If NOT KNOWN record 999)

H38. Loss of weight (days) __ __ __
____
(If NO record 000. If NOT KNOWN record 999)

H39. Night sweats (days) __ __ __
____
(If NO record 000. If NOT KNOWN record 999)

H40. Fever (days) __ __ __
____
(If NO record 000. If NOT KNOWN record 999)

Co-Morbid Conditions

H41. Diabetes
◯ No Diabetes
◯ Diabetes - Insulin injection
◯ Diabetes - Oral treatment only
◯ Diabetes - Not treated

H42. Hypertension
◯ No Hypertension
◯ Hypertension - Oral treatment only
◯ Hypertension - Not Treated

Investigations

H43. Sputum collected?	◯ Yes<br>◯ No
H43.1. Date of collection	________________________
H44. Time of collection	________________________
H44.1. AM/PM	◯ AM<br>◯ PM
H45. GeneXpert	◯ M Tuberculosis detected<br>◯ M Tuberculosis not detected<br>◯ Not done
H46. Date taken (GeneXpert)	________________________
H47. GeneXpert Rif result	◯ Rifampicin resistance detected<br>◯ Rifampicin resistance not detected<br>◯ Not done
H48. Sputum smear result	◯ Negative/Not seen<br>◯ Scanty<br>◯ 1+<br>◯ 2+<br>◯ 3+<br>◯ Not done
H49. Date taken (Sputum smear)	________________________
H50. Sputum culture result	◯ Positive for M tuberculosis<br>◯ Negative<br>◯ Contaminated or lab problem<br>◯ Not done
H51. Date taken (Sputum culture)	________________________

Drug Sensitivity from any result in file or in the lab for this episode of TB

H52. Isoniazid	◯ Resistant<br>◯ Sensitive<br>◯ Not done
H53. Rifampicin	◯ Resistant<br>◯ Sensitive<br>◯ Not done
H54 If resistant to Isoniazid and Rifampicin, resistant to second-line drugs?	◯ Yes<br>◯ No

H55. Resistant to ________________________________

H55. Resistant to ________________________________

H55. Resistant to ________________________________

H56. Fingerprick HGT __ __.__mmol/L ________________________________

H57. Did you draw blood for HbA1C?
◯ Yes
◯ No

H58. HbA1C result __.__% ________________________________

TST Testing

H59. TST placed ________________________________

H60. TST read ________________________________

H61. Induration?
◯ Yes
◯ No

H62. Diameter __ __mm ________________________________

H63. Pre-existing liver disease?
◯ Yes
◯ No

H64. History of severe alcohol use?
◯ Yes
◯ No

H65. Symptoms of burning feet and weakness in feet and legs?
◯ Yes
◯ No

H66. Was a prescription written?
◯ Yes
◯ No

Reason, prescription not written ________________________________

H67. Was 1 month of treatment dispensed?
◯ Yes
◯ No

Reason, treatment not dispensed ________________________________

H68. Date first dose started ________________________________

H69. HIV Rapid test done?	◯ Yes<br>◯ No
H70. Result	◯ Positive<br>◯ Negative<br>◯ Invalid
H71. Confirmatory test done?	◯ Yes<br>◯ No
H72. Result	◯ Positive<br>◯ Negative<br>◯ Invalid
H73. Was an ELISA done?	◯ Yes<br>◯ No
H74. Result	◯ Positive<br>◯ Negative<br>◯ Invalid
H75. Children under 18 months, had an HIV PCR?	◯ Yes<br>◯ No
H76. Result	◯ Positive<br>◯ Negative<br>◯ Invalid
H77. OraQuick?	◯ Yes<br>◯ No
H78. Result	◯ Positive<br>◯ Negative<br>◯ Invalid
H79. Blood drawn for ELISA?	◯ Yes<br>◯ No
H80. Result	◯ Positive<br>◯ Negative<br>◯ Invalid
H81. DNA PCR?	◯ Yes<br>◯ No
H82. Result	◯ Positive<br>◯ Negative<br>◯ Invalid
H83. Anonymous oral fluid?	◯ Yes<br>◯ No
H84. Result	◯ Positive<br>◯ Negative<br>◯ Invalid

H85. Final HIV status today

◯ Positive
◯ Negative
◯ Inconclusive (Must retest in two weeks)

H86. Referred for ART?

◯ Yes
◯ No

H87. Blood drawn for CD4

____

H89. CD4 result __ __ __ __ cells/mm3

____

Form Completion

H90. Date form H completed

____

H91. Name of person completing this form

____

Audit Trail

Name of Data Entry Person

____

Date of Data Entry

____
(Kindly change the date every time a change is made to the data)

Household Census At 3 Month Followup Form I

Participant ID ________________________________

Name ________________________________
(First 2 letters of First Name and first 2 letters of Last Name)

I01. Who provided information for the CRF?
◯ Index case
◯ Next-of-kin
◯ Parent/Legal guardian

I02. Child headed household
◯ Yes
◯ No

I03. Total number of household members __ __
____

I04. Name
____

I05. Study ID : [record_id]-
____

I06. Age
____

I06. Age in
◯ Years
◯ Months

I07. Gender
◯ Male
◯ Female

I08. Relationship to index
◯ Husband/Wife/Partner
◯ Son/Daughter/Stepchild
◯ Brother/Sister
◯ Parent (mother/father)
◯ Parent-in-law
◯ Grandparent
◯ Grandchild
◯ Other

I08.1. Describe other
____

I09. Confirm this person is alive
◯ Yes
◯ No

I10. Left this household since baseline census?
◯ Yes
◯ No
◯ N/A

I11. Does this person have TB symptoms now?	◯ Yes<br>◯ No<br>◯ N/A
I12. Cough __ __	______________________
I13. Weight loss __ __	______________________
I14. Fever __ __	______________________
I15. Night sweats __ __	______________________
I16. Is this person taking TB treatment now?	◯ Yes<br>◯ No<br>◯ N/A<br>◯ Don't know
I17. Is this person taking ART?	◯ Yes<br>◯ No<br>◯ N/A<br>◯ Don't know

New people living in the household

I18.01. Name	______________________
I19.01. Study ID : [record_id]-	______________________
I20.01. Age	______________________
I21.01. Gender	◯ Male<br>◯ Female
I22.01. Relationship to index	◯ Husband/Wife/Partner<br>◯ Son/Daughter/Stepchild<br>◯ Brother/Sister<br>◯ Parent (mother/father)<br>◯ Parent-in-law<br>◯ Grandparent<br>◯ Grandchild<br>◯ Other
I22.01.1. Describe other	______________________
I23.01. Is this person is alive	◯ Yes<br>◯ No
I24.01. New household member since baseline census?	◯ Yes<br>◯ No

Does this person have TB symptoms now?
◯ Yes
◯ No
◯ N/A

I25.01. Cough __ __
____

I26.01. Weight loss __ __
____

I27.01. Fever __ __
____

I28.01. Night sweats __ __
____

I29.01. Is this person taking TB treatment now?
◯ Yes
◯ No
◯ N/A
◯ Don't know

I30.01. Is this person taking ART?
◯ Yes
◯ No
◯ N/A
◯ Don't know

I18.02. Name
____

I19.02. Study ID : [record_id]-
____

I20.02. Age
____

I21.02. Gender
◯ Male
◯ Female

I22.02. Relationship to index
◯ Husband/Wife/Partner
◯ Son/Daughter/Stepchild
◯ Brother/Sister
◯ Parent (mother/father)
◯ Parent-in-law
◯ Grandparent
◯ Grandchild
◯ Other

I22.02.1. Describe other
____

I23.02. Is this person is alive
◯ Yes
◯ No

I24.02. New household member since baseline census?
◯ Yes
◯ No

Does this person have TB symptoms now?	◯ Yes<br>◯ No<br>◯ N/A
I25.02. Cough __ __	__________________________________
I26.02. Weight loss __ __	__________________________________
I27.02. Fever __ __	__________________________________
I28.02. Night sweats __ __	__________________________________
I29.02. Is this person taking TB treatment now?	◯ Yes<br>◯ No<br>◯ N/A<br>◯ Don't know
I30.02. Is this person taking ART?	◯ Yes<br>◯ No<br>◯ N/A<br>◯ Don't know
I18.03. Name	__________________________________
I19.03. Study ID : [record_id]-	__________________________________
I20.03. Age	__________________________________
I21.03. Gender	◯ Male<br>◯ Female
I22.03. Relationship to index	◯ Husband/Wife/Partner<br>◯ Son/Daughter/Stepchild<br>◯ Brother/Sister<br>◯ Parent (mother/father)<br>◯ Parent-in-law<br>◯ Grandparent<br>◯ Grandchild<br>◯ Other
I22.03.1. Describe other	__________________________________
I23.03. Is this person is alive	◯ Yes<br>◯ No
I24.03. New household member since baseline census?	◯ Yes<br>◯ No

Does this person have TB symptoms now?	◯ Yes<br>◯ No<br>◯ N/A
I25.03. Cough __ __	______________________
I26.03. Weight loss __ __	______________________
I27.03. Fever __ __	______________________
I28.03. Night sweats __ __	______________________
I29.03. Is this person taking TB treatment now?	◯ Yes<br>◯ No<br>◯ N/A<br>◯ Don't know
I30.03. Is this person taking ART?	◯ Yes<br>◯ No<br>◯ N/A<br>◯ Don't know
I18.04. Name	______________________
I19.04. Study ID : [record_id]-	______________________
I20.04. Age	______________________
I21.04. Gender	◯ Male<br>◯ Female
I22.04. Relationship to index	◯ Husband/Wife/Partner<br>◯ Son/Daughter/Stepchild<br>◯ Brother/Sister<br>◯ Parent (mother/father)<br>◯ Parent-in-law<br>◯ Grandparent<br>◯ Grandchild<br>◯ Other
I22.04.1. Describe other	______________________
I23.04. Is this person is alive	◯ Yes<br>◯ No
I24.04. New household member since baseline census?	◯ Yes<br>◯ No

Does this person have TB symptoms now?	◯ Yes<br>◯ No<br>◯ N/A
I25.04. Cough __ __	__________________
I26.04. Weight loss __ __	__________________
I27.04. Fever __ __	__________________
I28.04. Night sweats __ __	__________________
I29.04. Is this person taking TB treatment now?	◯ Yes<br>◯ No<br>◯ N/A<br>◯ Don't know
I30.04. Is this person taking ART?	◯ Yes<br>◯ No<br>◯ N/A<br>◯ Don't know

Deceased

I31. Did anyone who lived in this household die in the past year?	◯ Yes<br>◯ No
I32. How many people died in the last year? __ __	__________________
I33.1. Age at death __ __ years	__________________
I34.1. Gender	◯ Male<br>◯ Female
I35.1. Died with TB?	◯ Yes<br>◯ No<br>◯ N/A<br>◯ Don't know
I36.1. Died whilst taking TB treatment?	◯ Yes<br>◯ No<br>◯ N/A<br>◯ Don't know
I37.1. Did they ever test HIV-positive?	◯ Yes<br>◯ No<br>◯ N/A<br>◯ Don't know

I38.1. Taking ART at death?	◯ Yes<br>◯ No<br>◯ N/A<br>◯ Don't know
I33.2. Age at death __ __ years	__________________________
I34.2. Gender	◯ Male<br>◯ Female
I35.2. Died with TB?	◯ Yes<br>◯ No<br>◯ N/A<br>◯ Don't know
I36.2. Died whilst taking TB treatment?	◯ Yes<br>◯ No<br>◯ N/A<br>◯ Don't know
I37.2. Did they ever test HIV-positive?	◯ Yes<br>◯ No<br>◯ N/A<br>◯ Don't know
I38.2. Taking ART at death?	◯ Yes<br>◯ No<br>◯ N/A<br>◯ Don't know
I33.3. Age at death __ __ years	__________________________
I34.3. Gender	◯ Male<br>◯ Female
I35.3. Died with TB?	◯ Yes<br>◯ No<br>◯ N/A<br>◯ Don't know
I36.3. Died whilst taking TB treatment?	◯ Yes<br>◯ No<br>◯ N/A<br>◯ Don't know
I37.3. Did they ever test HIV-positive?	◯ Yes<br>◯ No<br>◯ N/A<br>◯ Don't know
I38.3. Taking ART at death?	◯ Yes<br>◯ No<br>◯ N/A<br>◯ Don't know

I33.4. Age at death __ __ years

____

I34.4. Gender

◯ Male
◯ Female

I35.4. Died with TB?

◯ Yes
◯ No
◯ N/A
◯ Don't know

I36.4. Died whilst taking TB treatment?

◯ Yes
◯ No
◯ N/A
◯ Don't know

I37.4. Did they ever test HIV-positive?

◯ Yes
◯ No
◯ N/A
◯ Don't know

I38.4. Taking ART at death?

◯ Yes
◯ No
◯ N/A
◯ Don't know

Form Completion

I39. Date form I completed

____

I40. Name of person completing this form

____

Audit Trail

Name of Data Entry Person

____

Date of Data Entry

____
(Kindly change the date every time a change is made to the data)

Month 3 Household Member Form J

Participant ID

____

Name

____
(First 2 letters of First Name and first 2 letters of Last Name)

Vital Status and TB Outcomes

J01. Is the household member alive?
- ◯ Yes
- ◯ No

J01.01 Age

____

J01.02. Age in
- ◯ Years
- ◯ Months

J02. Date of death

____

J03. Household member currently taking TB treatment?
- ◯ Yes
- ◯ No (but should be taking)
- ◯ Not required to take TB treatment (currently not taking)

J03.a. Showed the GREEN treatment card and study team confirmed current treatment?
- ◯ Yes
- ◯ No (but should be taking)

J04. Type of treatment?
- ◯ Rifafour
- ◯ Other

J04.a. Describe other

____

J05. Date last treatment dispensed

____

J06. If household member not taking treatment, what is the main reason for not taking treatment?
- ◯ Could not travel to clinic to get more treatment
- ◯ Clinic ran out of treatment
- ◯ Tired of medicines
- ◯ Felt healthy
- ◯ Felt too sick
- ◯ Side effects (treatment making participant sick)
- ◯ Did not have food
- ◯ Religious beliefs
- ◯ Started traditional medicine or traditional healing
- ◯ Doctor told participant to stop
- ◯ Other

J06.a. Describe other

____

ART

J07. Household member currently taking ART?
◯ Yes
◯ No (but should be taking)
◯ Not required to take ART (currently not taking)

J08. Showed current ART treatment card and study team confirmed current treatment?
◯ Yes
◯ No (but should be taking)

J09. Type of ART?
◯ FDC
◯ Other

J09.a. Describe other
____

J10. Date last ART treatment dispensed
____

J11. If household member not taking treatment, what is the main reason for not taking ART?
◯ Could not travel to clinic to get more treatment
◯ Clinic ran out of treatment
◯ Tired of medicines
◯ Felt healthy
◯ Felt too sick
◯ Side effects (treatment making participant sick)
◯ Did not have food
◯ Religious beliefs
◯ Started traditional medicine or traditional healing
◯ Doctor told participant to stop
◯ Only took ART while pregnant
◯ Other

J11.a. Describe other
____

IPT

J12. Household member currently taking IPT?
◯ Yes
◯ No (but should be taking)
◯ Not required to take IPT (currently not taking)

J13. Showed current IPT treatment card and study team confirmed current treatment?
◯ Yes
◯ No (but should be taking)

J14. Date IPT dispensed this month
____

J15. If household member not taking treatment, what is the main reason for not taking IPT?
◯ Could not travel to clinic to get more treatment
◯ Clinic ran out of treatment
◯ Tired of medicines
◯ Felt healthy
◯ Felt too sick
◯ Side effects (treatment making participant sick)
◯ Did not have food
◯ Religious beliefs
◯ Started traditional medicine or traditional healing
◯ Doctor told participant to stop
◯ Other

J15.a. Describe other

____

Diabetes

J16. Household member currently on diabetes medication?

- ◯ Yes
- ◯ No (but should be taking)
- ◯ Not required to take diabetes treatment (currently not taking)

J17. Type of diabetes treatment?

- ◯ Gliclazide
- ◯ Metformin
- ◯ Insulin
- ◯ Told not to have diabetes treatment
- ◯ Not known

J18. Date last diabetes treatment dispensed

____

J19. If not on diabetes treatment, HGT today: __ __.__mmol/L

____

J20. If household member not taking treatment, what is the main reason for not taking diabetes treatment?

- ◯ Could not travel to clinic to get more treatment
- ◯ Clinic ran out of treatment
- ◯ Tired of medicines
- ◯ Felt healthy
- ◯ Felt too sick
- ◯ Side effects (treatment making participant sick)
- ◯ Did not have food
- ◯ Religious beliefs
- ◯ Started traditional medicine or traditional healing
- ◯ Doctor told participant to stop
- ◯ Other

J20.a. Describe other

____

Hypertension

J21. Household member taking hypertension treatment?

- ◯ Yes
- ◯ No (but should be taking)
- ◯ Not required to take hypertension treatment (currently not taking)

J22. Type of hypertension treatment?

- ◯ Hydrochlorthiazide
- ◯ Other

J22.a. Describe other

____

J23. Date last hypertension treatment dispensed

____

J24.1. BP recording [1] __ __ __/__ __ __

____

J24.2. BP recording [2] __ __ __/__ __ __

____

J24.3. BP recording [3] __ __ __/__ __ __

____

J25. If household member not taking treatment, what is the main reason for not taking hypertension treatment?

- ◯ Could not travel to clinic to get more treatment
- ◯ Clinic ran out of treatment
- ◯ Tired of medicines
- ◯ Felt healthy
- ◯ Felt too sick
- ◯ Side effects (treatment making participant sick)
- ◯ Did not have food
- ◯ Religious beliefs
- ◯ Started traditional medicine or traditional healing
- ◯ Doctor told participant to stop
- ◯ Other

J25.a. Describe other

____

Symptom Screen

J26. Cough (days) __ __ __

____
(If NO record 000. If NOT KNOWN record 999)

J27. Productive cough (days) __ __ __

____
(If NO record 000. If NOT KNOWN record 999)

J28. Blood stained or bloody cough (days) __ __ __

____
(If NO record 000. If NOT KNOWN record 999)

J29. Loss of weight (days) __ __ __

____
(If NO record 000. If NOT KNOWN record 999)

J30. Night sweats (days) __ __ __

____
(If NO record 000. If NOT KNOWN record 999)

J31. Fever (days) __ __ __

____
(If NO record 000. If NOT KNOWN record 999)

J32. Other (days) __ __ __

____
(If NO record 000. If NOT KNOWN record 999)

J32.a. Describe other

____

J33. Weight today __ __ __kg

____

Investigations

J34. Sputum collected?	◯ Yes<br>◯ No
J35. Date of collection	________________
J36. Time of collection	________________
J36.1. AM/PM	◯ AM<br>◯ PM
J37. GeneXpert	◯ M Tuberculosis detected<br>◯ M Tuberculosis not detected<br>◯ Not done
J38. Date taken (GeneXpert)	________________
J39. GeneXpert Rif result	◯ Rifampicin resistance detected<br>◯ Rifampicin resistance not detected<br>◯ Not done
J40. Sputum smear result	◯ Negative/Not seen<br>◯ Scanty<br>◯ 1+<br>◯ 2+<br>◯ 3+<br>◯ Not done
J41. Date taken (Sputum smear)	________________
J42. Sputum culture result	◯ Positive for M tuberculosis<br>◯ Negative<br>◯ Contaminated or lab problem<br>◯ Not done
J43. Date taken (Sputum culture)	________________

Drug Sensitivity from any result in file or in the lab for this episode of TB

J44. Isoniazid	◯ Resistant<br>◯ Sensitive<br>◯ Not done
J45. Rifampicin	◯ Resistant<br>◯ Sensitive<br>◯ Not done
J46. If resistant to Isoniazid and Rifampicin, resistant to second-line drugs?	◯ Yes<br>◯ No

J47.1. Resistant to ______________________

J47.2. Resistant to ______________________

J47.3. Resistant to ______________________

Form Completion

J48. Date form J completed ______________________

J49. Name of person completing this form ______________________

Audit Trail

Name of Data Entry Person ______________________

Date of Data Entry ______________________
(Kindly change the date every time a change is made to the data)

Household Census at Month 15 Followup Form K-Default

Participant ID	______________________ (Format: 0-00-0000-00)
K01: This index case was recruited in	______________________
K02: Household was:	◯ Located<br>◯ Not Located
K03: Who provided information for the CRF	☐ Index Case<br>☐ Head of the household<br>☐ Other senior household member
K04: Child-headed household	◯ Yes<br>◯ No
K05: Number of household members	______________________
Please complete form "K - Household Visits"	
Please complete form "K - New Household Members"	
K34: Did anyone who lived in this house die in the past 15 months	◯ Yes<br>◯ No
K35: How many people died in the past 15 months?	______________________
Please complete form, "K-Household Deaths"	
K42: Housing type (check only one response)	◯ Brick/concrete house<br>◯ Apartment / flats<br>◯ Traditional hut/dwelling<br>◯ Garage<br>◯ 1 Room<br>◯ Shack<br>◯ Other
Specify Other	______________________
K43: How many DINING ROOMS are there in the household?	______________________
K44: How many KITCHENS are there in the household?	______________________
K45: How many BEDROOMS are there in the household?	______________________

K46: How many LIVING ROOMS are there in the household? ________________________________

K47: How many BATHROOMS/TOILETS are there in the household? ________________________________

K48: Other rooms Type 1 ________________________________

K48: How many [k48] are there in the household? ________________________________

K49: Other rooms Type 2 ________________________________

K49: How many [k49] are there in the household? ________________________________

K50: How many rooms are there in the household in total (Sum K43-K49) ________________________________

K51: How many windows are there in the household? ________________________________

K52: How many doors are there in the household that lead outside ________________________________

K53: What are your main sources of energy for COOKING

- ◯ Electricity
- ◯ Gas
- ◯ Wood
- ◯ Coal
- ◯ Paraffin
- ◯ Candles
- ◯ Other

(Primary)

Specify Other ________________________________

K53: What are your main sources of energy for COOKING

- ◯ Electricity
- ◯ Gas
- ◯ Wood
- ◯ Coal
- ◯ Paraffin
- ◯ Candles
- ◯ Other

(Secondary)

Specify Other ________________________________

K54: What are your main sources of energy for HEATING	◯ Electricity<br>◯ Gas<br>◯ Wood<br>◯ Coal<br>◯ Paraffin<br>◯ Candles<br>◯ Other<br>(Primary)
Specify Other	__________________________________
K54: What are your main sources of energy for HEATING	◯ Electricity<br>◯ Gas<br>◯ Wood<br>◯ Coal<br>◯ Paraffin<br>◯ Candles<br>◯ Other<br>(Secondary)
Specify Other	__________________________________
K55: What are your main sources of energy for LIGHTING	◯ Electricity<br>◯ Gas<br>◯ Wood<br>◯ Coal<br>◯ Paraffin<br>◯ Candles<br>◯ Other<br>(Primary)
Specify Other	__________________________________
K55: What are your main sources of energy for LIGHTING	◯ Electricity<br>◯ Gas<br>◯ Wood<br>◯ Coal<br>◯ Paraffin<br>◯ Candles<br>◯ Other<br>(Secondary)
Specify Other	__________________________________
K56: How many people smoke tobacco inside the household?<br>Total people:	__________________________________<br>(000 = none & 99 = Don't know)
K57: For how many hours a day is there someone smoking inside the household?	__________________________________

K58: Main source of water for drinking/cooking/cleaning	◯ Government piped water<br>◯ Borehole<br>◯ Spring<br>◯ Dam/pool/stagnant water<br>◯ River or stream<br>◯ Other
Specify Other	__________________________________
K59: If HH has access to piped water, what kind	◯ Piped water inside the dwelling<br>◯ Piped water inside the yard<br>◯ Piped water in a community stand < 100m of household<br>◯ Piped water within >100m of household but not inaccessible<br>◯ Piped water inaccessible
K60: Toilet facilities	◯ Flush toilet<br>◯ Chemical toilet<br>◯ Pit toilet<br>◯ Bucket toilet<br>◯ Other
Specify Other	__________________________________
K61: Does your household have any of the following and how many?	☐ Refrigerator (working)<br>☐ Radio (working)<br>☐ Phone/landline (working)<br>☐ Internet (working)<br>☐ Microwave (working)<br>☐ Cellphone (working)<br>☐ Smart phone (working)<br>☐ Computer (working)<br>☐ Washing machine (working)<br>☐ Satellite dish/DSTV (working)
K61: Refrigerator No.	__________________________________
K61: Radio No.	__________________________________
K61: Phone/landline No.	__________________________________
K61: Internet No.	__________________________________
K61: Microwave No.	__________________________________
K61: Cellphone No.	__________________________________
K61: Smart phone No.	__________________________________

K61: Computer No.	________________________________
K61: Washing machine No.	________________________________
K61: Satellite dish/DSTV No.	________________________________
K62: Does household have a vehicle? (Working) If yes, how many	◯ Yes<br>◯ No
K63: If Yes, Number of vehicles	________________________________
K64: If YES, is the vehicle more than 7 years old:	◯ Yes<br>◯ No
K65: Does household have a Television (working)	◯ Large, flat screen<br>◯ TV is at least 7 years old<br>◯ No TV
K66: Does household have a stove (working)	◯ Gas stove<br>◯ Electric stove<br>◯ Paraffin stove<br>◯ Wood stove or cook with wood
K67: In the last 15 months have members of the household bought any of the household goods and appliances we have been asking you about?<br>If YES, which items in the last 15 months:	☐ Refrigerator<br>☐ Radio<br>☐ TV<br>☐ Phone<br>☐ Car<br>☐ DSTV Dish<br>☐ Cellphone<br>☐ Smart phone<br>☐ Stove<br>☐ Microwave<br>☐ Computer<br>☐ Internet<br>☐ Other
Specify Other	________________________________
L68: If YES, how much has been spent on those items in the last 15 months	________________________________<br>(R (ZAR))
K69: In the last 15 months have members of the household bought any equipment for their work or business?	◯ Yes<br>◯ No
L70: If YES, which items in the last 15 months:	☐ Tools<br>☐ Car<br>☐ Farm animals<br>☐ Computer<br>☐ Other

Specify Other	______________________
L71: If YES, how much has been spent on those items in the last 15 months	______________________
K72: If the household has an emergency, how much money would the household have in its savings account for urgent expenses?	◯ No savings<br>◯ < R100<br>◯ R100 - R500<br>◯ R500 - 1000<br>◯ R1000 - R2000<br>◯ R2000 - 5000<br>◯ > R5000
K73: In the past 15 months, children in the household	◯ Are in or have completed tertiary education<br>◯ Are in or have completed high school<br>◯ Are in or have completed primary school<br>◯ Are in or have completed preschool/cheche<br>◯ No children
K74: Does anyone who lives in the household ever go to bed hungry because there is no food available	◯ Yes<br>◯ No
K75: If YES, how many days per week	______________________
K76: How many people in the household receive an income (including pensions and grants)?	______________________
K77: What is the total monthly income of the household for all people?	______________________
K78: Household occupational status<br>Has any other household member or person sending regular money lost or gained employment/earning ability in the last 15 months	◯ Yes<br>◯ No
Lost<br>Household member study ID number	______________________
Lost<br>Household member study ID number	______________________
Lost<br>Household member study ID number	______________________
Or<br>Number of person/s sending money	______________________
Gained<br>Household member study ID number	______________________
Gained<br>Household member study ID number	______________________
Gained<br>Household member study ID number	______________________

Or<br>Number of person/s sending money	________________________
K79: In the past 15 months, has the household added or renovated a room/s	◯ Yes<br>◯ No
K80: If YES, what was the building cost of this renovation	________________________<br>(R (ZAR))

HOUSEHOLD ILLNESS

K81: Has any household member not been able to earn money, or missed picking up grant or other money or payment because of an illness?	◯ Yes<br>◯ No
K82: If YES, Estimate how much money was not earned or collected due to illness	________________________<br>(R (ZAR))
K83: Did any household member borrow money for healthcare to cover costs due to an illness?	◯ Yes<br>◯ No<br>◯ Don't know
K84: If YES, how much	________________________<br>(R (ZAR))
K85: If YES, from whom was the money borrowed	◯ Family<br>◯ Neighbour<br>◯ Friend<br>◯ Boyfriend/Girlfriend<br>◯ Bank<br>◯ Stokvel<br>◯ Machonisa/Loan Shark<br>◯ Employer<br>◯ Didn't borrow<br>◯ Other
Specify Other	________________________
K86: Did any Household member have to sell/pawn an asset/ livestock to pay for healthcare?	◯ Yes<br>◯ No
K87: If YES, what assets/livestock?	________________________
K88: Has the household had to reduce spending because of a sickness affecting a household member/s?	◯ Yes<br>◯ No

K89: What did the household reduce spending on the most
1)

- 0. No reduction in spending
- 1. Food
- 2. Entertainment (DSTV or other/soccer matches etc)
- 3. Petrol/transport/taxi fare
- 4. School expenditure/uniforms/books/outings
- 5. Clothing
- 6. Alcohol/ other drinks
- 7. Airtime
- 8. Savings
- 9. Helper/Domestic
- 10. Payments on furniture, other household goods bought on instalments, car, other
- 11. Other

K89: What did the household reduce spending on the most
2)

- 0. No reduction in spending
- 1. Food
- 2. Entertainment (DSTV or other/soccer matches etc)
- 3. Petrol/transport/taxi fare
- 4. School expenditure/uniforms/books/outings
- 5. Clothing
- 6. Alcohol/ other drinks
- 7. Airtime
- 8. Savings
- 9. Helper/Domestic
- 10. Payments on furniture, other household goods bought on instalments, car, other
- 11. Other

K90: On what other things did they reduce spending?
1)

- 0. No reduction in spending
- 1. Food
- 2. Entertainment (DSTV or other/soccer matches etc)
- 3. Petrol/transport/taxi fare
- 4. School expenditure/uniforms/books/outings
- 5. Clothing
- 6. Alcohol/ other drinks
- 7. Airtime
- 8. Savings
- 9. Helper/Domestic
- 10. Payments on furniture, other household goods bought on instalments, car, other
- 11. Other

K90: On what other things did they reduce spending?
2)

- 0. No reduction in spending
- 1. Food
- 2. Entertainment (DSTV or other/soccer matches etc)
- 3. Petrol/transport/taxi fare
- 4. School expenditure/uniforms/books/outings
- 5. Clothing
- 6. Alcohol/ other drinks
- 7. Airtime
- 8. Savings
- 9. Helper/Domestic
- 10. Payments on furniture, other household goods bought on instalments, car, other
- 11. Other

K90: On what other things did they reduce spending? 3)	◯ 0. No reduction in spending<br>◯ 1. Food<br>◯ 2. Entertainment (DSTV or other/soccer matches etc)<br>◯ 3. Petrol/transport/taxi fare<br>◯ 4. School expenditure/uniforms/books/outings<br>◯ 5. Clothing<br>◯ 6. Alcohol/ other drinks<br>◯ 7. Airtime<br>◯ 8. Savings<br>◯ 9. Helper/Domestic<br>◯ 10. Payments on furniture, other household goods bought on instalments, car, other<br>◯ 11. Other
K91: If someone in the household died, how much did funeral arrangements cost this household	☐ Did not have a funeral
K91: Amount	________________________________<br>(R (ZAR))
K92: On a scale of 1 to 5, in which 1 is no impact and 5 is very serious impact, to what extent has the TB illness affected the family costs and income?	◯ No impact<br>◯ Little impact<br>◯ Moderate impact<br>◯ Serious impact<br>◯ Very serious impact
K93: Have any HH members been sent to other households because yours could not cope since the illness started?	◯ Yes<br>◯ No
K94: If YES, How many members	________________________________
K95: Have any learners/ or students in the household dropped out from learning since the illness started?	◯ Yes<br>◯ No
K94: If YES, How many members	________________________________
K97: Date form completed	________________________________
K98: Name of person completing this form	________________________________

Audit Trail

Name of Data Entry Person	________________________________
Date of Data Entry	________________________________<br>(Kindly change the date every time a change is made to the data)

Household Census at Month 15 Form K-Household Visits

Participant ID	____________________ (Format: 0-00-0000-00)
K06: Name	____________________
K06: Surname	____________________
K07: Study ID	____________________
K08: Age	◯ Years ◯ Months
K08: Age	____________________
K09: Sex	◯ Male ◯ Female
K10: Is this person alive	◯ 1=Yes<br>◯ 2=No
K11: Left this household in the past 15 months	◯ 1=Yes<br>◯ 2=No<br>◯ 3=NA
K12: Does this person have TB symptoms now?	◯ Yes<br>◯ No
K13: Cough (of any duration)	◯ 1. Present<br>◯ 2. Not present<br>◯ 3. Not applicable
K14: Weight loss	◯ 1. Present<br>◯ 2. Not present<br>◯ 3. Not applicable
K15: Fever	◯ 1. Present<br>◯ 2. Not present<br>◯ 3. Not applicable
K16: Night sweats	◯ 1. Present<br>◯ 2. Not present<br>◯ 3. Not applicable
K17: Is this person taking TB treatment?	◯ 1=Yes<br>◯ 2=No<br>◯ 3=NA<br>◯ 4=Don't know

K18: Is this person taking ART	◯ 1=Yes<br>◯ 2=No<br>◯ 3=NA<br>◯ 4=Don't know
K19: Has participant consented to be included in the study	◯ 1=Yes<br>◯ 2=No

Audit Trail

Name of Data Entry Person	__________________________________
Date of Data Entry	__________________________________<br>(Kindly change the date every time a change is made to the data)

Household Census at Month 15 Followup Form K-New Household Members

Participant ID	______________________ (Format: 0-00-0000-00)
K20: Name	______________________
K20: Surname	______________________
K21: Study ID	______________________
K22: Age	◯ Years ◯ Months
K22: Age	______________________
K23: Sex	◯ Male ◯ Female
K24: Relationship to Index	◯ 1=Husband/wife/partner<br>◯ 2=Son/daughter/stepchild<br>◯ 3=Brother/sister<br>◯ 4=Parent(mother/father)<br>◯ 5=Parent-in-law<br>◯ 6=Grandparent<br>◯ 7=Grandchild<br>◯ 8=Other
K25: Is this person alive	◯ 1=Yes<br>◯ 2=No
K26: New Household member entered in past 15 months	◯ 1=Yes<br>◯ 2=No<br>◯ 3=NA
K26: Date they started living	______________________

Does this person have TB symptoms now?

K27: Cough (of any duration)	◯ 1. Present<br>◯ 2. Not Present<br>◯ 3. Not applicable
K28: Weight loss	◯ 1. Present<br>◯ 2. Not Present<br>◯ 3. Not applicable
K29: Fever	◯ 1. Present<br>◯ 2. Not Present<br>◯ 3. Not applicable

K30: Night sweats
- ◯ 1. Present
- ◯ 2. Not Present
- ◯ 3. Not applicable

K31: Has this person taken TB treatment in the last 15 months
- ◯ 1=Yes
- ◯ 2=No
- ◯ 3=NA
- ◯ 4=Don't know

K31: Start date of TB treatment

____

K32: Is this person taking ART
- ◯ 1=Yes
- ◯ 2=No
- ◯ 3=NA
- ◯ 4=Don't know

K33: Has participant consented to be included in the study
- ◯ 1=Yes
- ◯ 2=No

Audit Trail

Name of Data Entry Person

____

Date of Data Entry

____
(Kindly change the date every time a change is made to the data)

Household Census at Month 15 Followup Form
K-Household Deaths

Participant ID

____
(Format: 0-00-0000-00)

K36: Age at death

____

K37: Gender

◯ Male
◯ Female

K38: Date of death (estimate date if not known)

____

K38: Died with likely TB, not on treatment?

◯ Yes
◯ No
◯ Don't know

K39: Died whilst taking TB treatment?

◯ Yes
◯ No
◯ Don't know

K40: Did they ever test HIV-positive?

◯ Yes
◯ No
◯ Don't know

K41: Taking ART at death?

◯ Yes
◯ No
◯ Don't know

Audit Trail

Name of Data Entry Person

____

Date of Data Entry

____
(Kindly change the date every time a change is made to the data)

Month 15 Household Member Form L

i. Household was	◯ Identified<br>◯ Not identified
ii. Date of Interview	__________________________________
iii. Participant ID:<br>[d5]	__________________________________
iv. Name :<br>[d4]	__________________________________<br>(First 2 letters of First Name and first 2 letters of Last Name)
v. Age:<br>[d6]	__________________________________
v. Age in	◯ Years<br>◯ Months
vi. Who was interviewed to get data on this household member?	◯ This household member<br>◯ Another household member/Index case from this household<br>◯ Someone else(specify)
vi. Specify	__________________________________
vii. Household member was:	◯ Identified<br>◯ Not able to be contacted

Health Information

L01. Is the household member alive today?	◯ Yes<br>◯ No
L02. Death confirmed by	◯ Inspection of Death certificate<br>◯ Verbal report (from whom)<br>◯ Other (provide details)
L02.01. Verbal report from:	__________________________________
L02.02. Other, provide details:	__________________________________
L03. Date of death	__________________________________

L04. If not alive what was the cause of death? (select all that apply.)<br>Verbal response from family member is fine	☐ TB<br>☐ HIV<br>☐ Diarrhoea<br>☐ Pneumonia<br>☐ Meningitis<br>☐ Accident(vehicle or other)<br>☐ Violence(stab, gunshot, blunt etc.)<br>☐ Stroke<br>☐ Other
L04.01. Describe other	______________________
L05. Weight (no shoes or jacket) if person is available	______________________<br>(__ __ __kg)
L06. Height if younger than 18yrs	______________________<br>(__ __ __cm)
L07. Blood pressure	______________________<br>(__ __ __/__ __ __ (record the 3rd reading after 2 prior readings))

If in third reading, systolic BP>140 or diastolic BP>90, then refer to the nearest clinic for assessment or review of hypertension

L08. MUAC	______________________<br>(__ __mm)

HIV/ART

L09. Has the participant ever been tested for HIV?	◯ Yes<br>◯ No
L10. HIV status before this visit HIV testing	◯ Positive for HIV<br>◯ Negative (must offer HIV test at this visit)<br>◯ Unable to infer HIV status (must offer HIV test at this visit)
L11. Has the participant tested for HIV in the last 15 months?	◯ Yes<br>◯ No
L12. What was the result of the participant's most recent HIV test?	◯ Positive for HIV<br>◯ Negative<br>◯ Invalid<br>◯ Not tested

~If HIV-Positive~

L13. Is participant currently taking ART drugs for treatment of HIV?	◯ Yes<br>◯ No
L14. Showed current ART treatment card and study team confirmed current treatment?	◯ Yes<br>◯ No
L15. Type of ART?	◯ FDC<br>◯ Other
L15.01. Describe other	__________________________________
L16. When did you first start ART?<br>(estimate is OK)	__________________________________
L17. If participant is HIV positive, but not taking ART, what is the main reason for not taking ART?	◯ Could not travel to clinic to get more treatment<br>◯ Clinic ran out of treatment<br>◯ Tired of medicines<br>◯ Felt healthy<br>◯ Felt too sick to take ART<br>◯ Side effects (treatment making participant sick)<br>◯ Did not have food<br>◯ Religious beliefs<br>◯ Started traditional medicine or traditional healing<br>◯ Doctor told participant to stop<br>◯ Only took ART while pregnant<br>◯ Other
L17.01. Describe other	__________________________________
L18. Has participant ever taken ART drugs for treatment of HIV?	◯ Yes<br>◯ No
L19. Last date ART was dispensed to this patient?	__________________________________

~If HIV-Negative~

L20. HIV Rapid test done at this study visit?	◯ Yes<br>◯ No
L21. Result of rapid HIV test	◯ Positive<br>◯ Negative<br>◯ Invalid
L22. If rapid test Positive, result of confirmatory rapid test	◯ Positive for HIV<br>◯ Negative<br>◯ Invalid
L23. If discordant or invalid was an ELISA taken?	◯ Yes<br>◯ No
L24. Children younger 18 months, had an HIV PCRat this final study visit?	◯ Yes<br>◯ No

L25. OraQuick done at this final study visit?

◯ Yes
◯ No

L26. Lab result of oral fluid (OraQuick)

◯ Positive for HIV
◯ Negative
◯ Invalid

L27. Anonymous oral fluid taken at this final study visit?

◯ Yes
◯ No

L28. Lab result of oral fluid (Anonymous oral fluid)

◯ Positive for HIV
◯ Negative
◯ Invalid

Final Study HIV Status

L29. Final HIV status of this participant using all results and all self-reports

◯ Positive for HIV
◯ Negative
◯ Inconclusive (Must retest in two weeks)
◯ Not tested

If HIV Positive, draw blood for CD4 count and complete Investigation section

TB Disease and Treatment

L30. In the past 15 months, has this household member been diagnosed with TB; or, has this household member been prescribed (or told) to take TB treatment?

(Include any TB diagnosed in index cases, and also TB in HH members diagnosed by the study)

◯ Yes
◯ No

L31. If yes how many episodes was this person told s/he had TB in the past 15 months?

◯ One
◯ Two
◯ More than two

L32. If yes to TB episode, date that the participant was first told that s/he had TB

____

L33. If YES to TB in the past 15 months, did the study team see a source document that confirmed TB including those diagnosed by the study at baseline?

(tick all that apply)

☐ Green clinic TB card
☐ Lab result Xpert or culture: M TB detected
☐ TB register or other clinic document at local clinic
☐ No document able to be found that confirms TB

L34. Date this specimen confirming TB diagnosis for episode 1 was collected?

____

L35. If YES to TB in past 15 months, date first dose of TB treatment taken/started

____

L36. Date TB treatment completed

____

L36. or,

☐ Currently taking TB treatment

L37. If YES to TB in past 15 months , what treatment was used in the first two months of TB treatment phase ?<br>(select all that apply)	☐ Rifafor (or equivalent four drug single dose tablet/s)<br>☐ A regimen that includes at least three of these TB drugs: Clofazamine; either amikacin or kanamycin; bedaquilin; levofloxacin or moxifloxacin; ethionamide<br>☐ Other regimen
L37.1. Describe other	__________________________

Episode 2

L38. Did the participant have a second episode of TB in the past 15 months?	◯ Yes<br>◯ No
L39. If YES to second episode of TB, date the participant was first told that s/he had the second episode of TB<br>(Please estimate if no source document available)	__________________________
L40. If YES to second episode of TB, did the study team see a source document that confirmed TB including those diagnosed by the study at baseline	☐ Green clinic TB card<br>☐ Lab result Xpert or culture: M TB detected<br>☐ TB register or other clinic document at local clinic<br>☐ No document able to be found that confirms TB
L41. Date this specimen confirming TB diagnosis for episode 2 was collected/taken?	__________________________
L42. If YES to second episode of TB, what type of TB based on resistance patterns	◯ Drug sensitive (no resistance detected)<br>◯ Rifampicin resistance<br>◯ MDR (Rifampicin and INH resistance)<br>◯ XDR
L43. If YES to second episode of TB in past 15 months, date TB treatment first dose taken/started	__________________________
L44. Date TB treatment completed	__________________________
L44. or,	☐ Currently taking TB treatment

Isoniazid Preventive Therapy (IPT)

L45. Did participant ever take any form of preventive therapy IPT or 3HP either prescribed by the study or a clinic?	◯ Yes<br>◯ No
L46. If yes did study team see some written confirmation of IPT treatment?	◯ Yes<br>◯ No
L47. Date IPT started	__________________________

L48. Estimate how many months of IPT the participant took over the past 15 months

__________________________ (months)

L49. If took fewer than six months of IPT, what main reason was given for stopping IPT?

- ◯ Could not travel to clinic to get more treatment
- ◯ Clinic ran out of IPT or refused to give IPT
- ◯ Tired of IPT
- ◯ Felt healthy
- ◯ Felt too sick to take IPT
- ◯ Side effects (treatment making participant sick)
- ◯ Did not have food
- ◯ Religious beliefs
- ◯ Started traditional medicine or traditional healing
- ◯ Doctor told participant to stop
- ◯ Other

L49.1. Describe other

____

Symptom Screen, Today

L50. Cough (days) __ __ __

__________________________ (If NO record 000. If NOT KNOWN record 999)

L51. Productive cough (days) __ __ __

__________________________ (If NO record 000. If NOT KNOWN record 999)

L52. Blood stained or bloody cough (days) __ __ __

__________________________ (If NO record 000. If NOT KNOWN record 999)

L53. Loss of weight (days) __ __ __

__________________________ (If NO record 000. If NOT KNOWN record 999)

L54. Night sweats (days) __ __ __

__________________________ (If NO record 000. If NOT KNOWN record 999)

L55. Fever (days) __ __ __

__________________________ (If NO record 000. If NOT KNOWN record 999)

L56. Other symptom

____

L56. [I56] (days) __ __ __

__________________________ (If NO record 000. If NOT KNOWN record 999)

If any symptom of TB, collect a sputum sample and complete Investigation section

Health Economics

L57. Nature of illness

- ☐ HIV
- ☐ TB
- ☐ No illness
- ☐ Other

L57.01. Describe other

____

L58. Date the participant started being sick or got diagnosed

____

L59. Member's occupational status before illness

- ◯ Formal employ (full-time)
- ◯ Formal employ (part-time)
- ◯ Self-employed
- ◯ Informal
- ◯ Piece jobs
- ◯ Student - FET/Tertiary
- ◯ In-school learner
- ◯ Pension
- ◯ Unemployed - Looking for work
- ◯ Unemployed - Not looking for work
- ◯ Pre-school going
- ◯ Other

L59.01. Describe other

____

L60. Member's occupation status now

- ◯ Formal employ (full-time)
- ◯ Formal employ (part-time)
- ◯ Self-employed
- ◯ Informal
- ◯ Piece jobs
- ◯ Student - FET/Tertiary
- ◯ In-school learner
- ◯ Pension
- ◯ Unemployed - Looking for work
- ◯ Unemployed - Not looking for work
- ◯ Pre-school going
- ◯ Other

L60.01. Describe other

____

~Visits made for medical help related to that new illness~

L61.01. Visits to primary care clinic

____
(__ __ visits)

L61.02. Visits to community health centre

____
(__ __ visits)

L61.03. Visits to TB clinic

____
(__ __ visits)

L61.04. Visits to VCT centre

____
(__ __ visits)

L61.05. Visits to hospital

____
(__ __ visits)

L61.06. Visits to GP

____
(__ __ visits)

L61.07. Visits to pharmacist

____
(__ __ visits)

L61.08. Visits to traditional healer

____
(__ __ visits)

L61.09. Visits to "other"

____
(__ __ visits)

L61.09.01. Describe other

____

L62. Was the participant admitted to hospital(s) for the illness in the past 15 months?

◯ Yes
◯ No

L62.01.01. Hospital [1st admission]

____

L62.01.02. Number of nights stay [1st admission]

____

L62.01.03 Date of admission [1st admission]

____

L62.02.01. Hospital [2nd admission]

____

L62.02.02. Number of nights stay [2nd admission]

____

L62.02.03 Date of admission [2nd admission]

____

L62.03.01. Hospital [3rd admission]

____

L62.03.02. Number of nights stay [3rd admission]

____

L62.03.03 Date of admission [3rd admission]

____

L62.04. Total number of admissions if more that 3 hospital admissions

____

L63. How does the participant usually travel to the hospital or clinic to get care?

- ◯ Private car
- ◯ Minibus or other taxi
- ◯ Bus
- ◯ Train
- ◯ Walk
- ◯ Other

L63.01. Describe other

____

L64. What is the cost of one trip to get care?
R

____

Quality of Life

L65. What do you expect will happen to the quality of life for the people in this household over the next year?

- ◯ Much better than 1 year ago
- ◯ Somewhat better than 1 year ago
- ◯ About the same
- ◯ Somewhat worse than 1 year ago
- ◯ Much worse than 1 year ago

L66. In general your health is

- ◯ Excellent
- ◯ Very good
- ◯ Good
- ◯ Fair
- ◯ Poor

Investigations for this visit

L67. Date blood was collected for an HIV ELISA

____

L68. Result of ELISA

- ◯ Positive for HIV
- ◯ Negative

L69. Date sample was collected for children < 18 months for an HIV PCR

____

L70. Lab result for HIV PCR

- ◯ Positive for HIV
- ◯ Negative

L71. Date blood drawn for CD4 count

____

L72. Laboratory CD4 result

____
(__ __ __ __cells/mm3)

Investigations done on this participant

L73. Sputum collected?<br>(Sputum must be collected if participant reports any TB symptom)	◯ Yes<br>◯ No
L74. Date of sputum collection<br>(must be atleast 4ml)	______________________
L75. Time of sputum collection	______________________
L76. GeneXpert result of this sputum	◯ M Tuberculosis detected<br>◯ M Tuberculosis not detected<br>◯ Not done
L77. GeneXpert Rif result if M Tuberculosis detected	◯ Rifampicin resistance detected<br>◯ Rifampicin resistance not detected
L78. Sputum smear result	◯ Negative/Not seen<br>◯ Scanty<br>◯ 1+<br>◯ 2+<br>◯ 3+<br>◯ Not done
L79. Sputum culture result	◯ Positive for M tuberculosis<br>◯ Negative<br>◯ Contaminated or lab problem<br>◯ Not done
L80. If sputum culture result was contaminated or lab problem, was a second sputum sample collected?	◯ Yes<br>◯ No
L81. Date of second sputum collection<br>(must be atleast 4ml)	______________________
L82. Time of second sputum collection	______________________
L83. Second sputum culture result	◯ Positive for M tuberculosis<br>◯ Negative<br>◯ Contaminated or lab problem<br>◯ Not done
L84. If second sputum culture result was contaminated or lab problem, was a third sputum sample collected?	◯ Yes<br>◯ No
L85. Date of third sputum collection<br>(must be atleast 4ml)	______________________
L86. Time of third sputum collection	______________________

L87. Third sputum culture result

◯ Positive for M tuberculosis
◯ Negative
◯ Contaminated or lab problem
◯ Not done

Drug Sensitivity from any result in file or in the lab for this episode of TB

L88. Isoniazid

◯ Resistant
◯ Sensitive
◯ Not done

L89. Rifampicin

◯ Resistant
◯ Sensitive
◯ Not done

TST Testing (only for children 14 years and younger)

L90. TST placed?

◯ Yes
◯ No

L91. Date TST placed

____

L92. Date TST read

____

L92. or,

☐ TST not read

L93. Induration?

◯ Yes
◯ No

L94. Transverse diameter of induration __ __mm

____

Other Investigations

L95. Finger prick HGT

____
(mmol/L)

L96. Did you draw blood for HbA1c?

◯ Yes
◯ No

L97. HbA1c result

____
(%)

If more than 5.5%, refer for treatment with results

L98. Diabetes

◯ No diabetes
◯ Diabetes - Insulin injection
◯ Diabetes - Oral treatment only
◯ Diabetes - Not treated

L99. Hypertension

◯ No hypertension
◯ Hypertension - Oral treatment only
◯ Hypertension - Not treated

Form Completion

L100. Date Form L finally completed (including all Lab results) __________________________________

L101. Initials of person who did visual review __________________________________

Audit Trail

Name of Data Entry Person __________________________________

Date of Data Entry __________________________________
(Kindly change the date every time a change is made to the data)

Untoward Household Event Form M

M01. Date of Contact

____

M02. Contact person

____

M03. Date event happened

____

M04. Time event happened

____

M04.1. AM/PM

- ◯ AM
- ◯ PM

M04.2. Time of day

- ◯ Morning
- ◯ Afternoon
- ◯ Evening

M05. Status of event

- ◯ New
- ◯ Ongoing

M06. Location where event occured

- ◯ At the household
- ◯ Neighbours house
- ◯ In the community
- ◯ At the clinic/hospital
- ◯ At the shops
- ◯ Other

M06.1. Describe other

____

M07. Tick all people that were involved in the event that is being reported

- ☐ Husband/Wife/Partner
- ☐ Son/Daughter/Stepchild
- ☐ Brother/Sister
- ☐ Parent (mother/father)
- ☐ Parent-in-law
- ☐ Grandparent
- ☐ Grandchild
- ☐ Neighbours
- ☐ Community members
- ☐ Friends
- ☐ Study staff
- ☐ Other

M07.1. Describe other

____

M08. Summary of event	☐ Verbal assault<br>☐ Physical assault<br>☐ Adverse drug event (INH related)<br>☐ Adverse drug event (Non-INH related)<br>☐ Suicide attempt<br>☐ Police intervention<br>☐ Job loss<br>☐ Kicked out of home<br>☐ Stigmatization<br>☐ Other
M08.1. Describe other	__________________________________
M09. Provide a detailed description of the event	____
M10. Has study PI been informed about the event?	◯ Yes<br>◯ No
M11. Describe the action that has been taken following the event	____
M12. Is the event that is being reported related to the intervention?	◯ Yes<br>◯ No<br>◯ Unclear
M13. Outcome	◯ Resolved<br>◯ Ongoing

Form Completion

M14. Date form M completed	__________________________________
M15. Name of person completing this form	__________________________________

Audit Trail

Name of Data Entry Person	__________________________________
Date of Data Entry	__________________________________<br>(Kindly change the date every time a change is made to the data)

Study Termination Form N

Participant ID	______________________
Name	______________________ (First 2 letters of First Name and first 2 letters of Last Name)
N01. Person terminated from study	◯ Index case<br>◯ Household member
N01.1. Household member Participant ID	______________________
N02. Date of termination	______________________
N03. Participant terminated due to	◯ Death (died during study period)<br>◯ Lost to follow-up<br>◯ Relocated<br>◯ Following PI's decision<br>◯ Participant/Next-of-kin/Legal guardian withdrew consent<br>◯ Other
N04. Provide reason for withdrawal of consent	______________________
N05. Describe other	______________________

Form Completion

N06. Date form N completed	______________________
N07. Name of person completing this form	______________________

Audit Trail

Name of Data Entry Person	______________________
Date of Data Entry	______________________ (Kindly change the date every time a change is made to the data)

Health Economics Form HE

Form completed at:	◯ Baseline<br>◯ Month 3
i. Start Time	__________________________________

Baseline

ii. Date of Interview	__________________________________
iii. Status of Index Case	◯ Alive<br>◯ Deceased
iii.i. Date decesaed	__________________________________
iv. Date of TB diagnosis	__________________________________
v. Who is the main person providing information for this questionnaire?	◯ Index case<br>◯ Other
v.i. Relation to Index case	◯ Wife/Mother<br>◯ Husband/Father<br>◯ Extended family<br>◯ Son/Daughter<br>◯ Other
v.i.i. Describe other	__________________________________

Household Socio-economic Status

HE01. How many people in the household receive an income?<br>(including pensions and grants)	__________________________________
HE02. Who is the primary income earner in the household?	◯ Index<br>◯ Wife/Mother<br>◯ Husband/Father<br>◯ Extended family<br>◯ Son/Daughter<br>◯ Other
HE02.1 Describe other	__________________________________

HE03. Does your household have any of the following and how many?

- ☐ Refrigerator
- ☐ Stove
- ☐ Television
- ☐ Car
- ☐ Radio
- ☐ Phone-landline
- ☐ Internet
- ☐ Microwave
- ☐ Cellphone
- ☐ Smartphone
- ☐ Computer
- ☐ Washing machine
- ☐ Satellite dish/DSTV

HE03.01. Number - Refrigirator ______________________

HE03.02. Number - Stove ______________________

HE03.03. Number - Television ______________________

HE03.04. Number - Car ______________________

HE03.05. Number - Radio ______________________

HE03.06. Number - Phone-landline ______________________

HE03.07. Number - Internet ______________________

HE03.08. Number - Microwave ______________________

HE03.09. Number - Cellphone ______________________

HE03.10. Number - Smartphone ______________________

HE03.11. Number - Computer ______________________

HE03.12. Number - Washing machine ______________________

HE03.13. Number - Satellite dish/DSTV ______________________

HE04. Household food production

- ☐ Livestock
- ☐ Mielies/Sorghum
- ☐ Vegetables
- ☐ Poultry
- ☐ Other

HE04.1. Describe other

____

HE05. Do you have additional income sent by household members not living with you?

- ◯ Yes
- ◯ No

HE05.1. How many?

____

HE06. Amount.R-

____

HE07. Household members

____

Household member - 01

HE08. Occupation status

- ◯ Formal employ (full-time)
- ◯ Formal employ (part-time)
- ◯ Self-employed
- ◯ Informal
- ◯ Piece jobs
- ◯ Student - FET/Tertiary
- ◯ In-school learner
- ◯ Pension
- ◯ Unemployed - Looking for work
- ◯ Unemployed - Not looking for work
- ◯ Pre-school going
- ◯ Other

HE08.1 Describe other

____

HE09. Amount earned per month

- ◯ R0
- ◯ R1-R999
- ◯ R1000-R4999
- ◯ R5000-R9999
- ◯ R10000+

HE10. Source

- ◯ Wages/Income
- ◯ Pension
- ◯ Disability grant
- ◯ Child grant
- ◯ Other

HE10.1 Describe other

____

Household member - 02

HE08. Occupation status

- ◯ Formal employ (full-time)
- ◯ Formal employ (part-time)
- ◯ Self-employed
- ◯ Informal
- ◯ Piece jobs
- ◯ Student - FET/Tertiary
- ◯ In-school learner
- ◯ Pension
- ◯ Unemployed - Looking for work
- ◯ Unemployed - Not looking for work
- ◯ Pre-school going
- ◯ Other

HE08.1 Describe other

____

HE09. Amount earned per month

- ◯ R0
- ◯ R1-R999
- ◯ R1000-R4999
- ◯ R5000-R9999
- ◯ R10000+

HE10. Source

- ◯ Wages/Income
- ◯ Pension
- ◯ Disability grant
- ◯ Child grant
- ◯ Other

HE10.1 Describe other

____

Household member - 03

HE08. Occupation status

- ◯ Formal employ (full-time)
- ◯ Formal employ (part-time)
- ◯ Self-employed
- ◯ Informal
- ◯ Piece jobs
- ◯ Student - FET/Tertiary
- ◯ In-school learner
- ◯ Pension
- ◯ Unemployed - Looking for work
- ◯ Unemployed - Not looking for work
- ◯ Pre-school going
- ◯ Other

HE08.1 Describe other

____

HE09. Amount earned per month

- ◯ R0
- ◯ R1-R999
- ◯ R1000-R4999
- ◯ R5000-R9999
- ◯ R10000+

HE10. Source	◯ Wages/Income<br>◯ Pension<br>◯ Disability grant<br>◯ Child grant<br>◯ Other
HE10.1 Describe other	__________________________________

Household member - 04

HE08. Occupation status	◯ Formal employ (full-time)<br>◯ Formal employ (part-time)<br>◯ Self-employed<br>◯ Informal<br>◯ Piece jobs<br>◯ Student - FET/Tertiary<br>◯ In-school learner<br>◯ Pension<br>◯ Unemployed - Looking for work<br>◯ Unemployed - Not looking for work<br>◯ Pre-school going<br>◯ Other
HE08.1 Describe other	__________________________________
HE09. Amount earned per month	◯ R0<br>◯ R1-R999<br>◯ R1000-R4999<br>◯ R5000-R9999<br>◯ R10000+
HE10. Source	◯ Wages/Income<br>◯ Pension<br>◯ Disability grant<br>◯ Child grant<br>◯ Other
HE10.1 Describe other	__________________________________

Household member - 05

HE08. Occupation status	◯ Formal employ (full-time)<br>◯ Formal employ (part-time)<br>◯ Self-employed<br>◯ Informal<br>◯ Piece jobs<br>◯ Student - FET/Tertiary<br>◯ In-school learner<br>◯ Pension<br>◯ Unemployed - Looking for work<br>◯ Unemployed - Not looking for work<br>◯ Pre-school going<br>◯ Other
HE08.1 Describe other	__________________________________

HE09. Amount earned per month

- ◯ R0
- ◯ R1-R999
- ◯ R1000-R4999
- ◯ R5000-R9999
- ◯ R10000+

HE10. Source

- ◯ Wages/Income
- ◯ Pension
- ◯ Disability grant
- ◯ Child grant
- ◯ Other

HE10.1 Describe other

____

Household member - 06

HE08. Occupation status

- ◯ Formal employ (full-time)
- ◯ Formal employ (part-time)
- ◯ Self-employed
- ◯ Informal
- ◯ Piece jobs
- ◯ Student - FET/Tertiary
- ◯ In-school learner
- ◯ Pension
- ◯ Unemployed - Looking for work
- ◯ Unemployed - Not looking for work
- ◯ Pre-school going
- ◯ Other

HE08.1 Describe other

____

HE09. Amount earned per month

- ◯ R0
- ◯ R1-R999
- ◯ R1000-R4999
- ◯ R5000-R9999
- ◯ R10000+

HE10. Source

- ◯ Wages/Income
- ◯ Pension
- ◯ Disability grant
- ◯ Child grant
- ◯ Other

HE10.1 Describe other

____

Household member - 07

HE08. Occupation status

- ◯ Formal employ (full-time)
- ◯ Formal employ (part-time)
- ◯ Self-employed
- ◯ Informal
- ◯ Piece jobs
- ◯ Student - FET/Tertiary
- ◯ In-school learner
- ◯ Pension
- ◯ Unemployed - Looking for work
- ◯ Unemployed - Not looking for work
- ◯ Pre-school going
- ◯ Other

HE08.1 Describe other

____

HE09. Amount earned per month

- ◯ R0
- ◯ R1-R999
- ◯ R1000-R4999
- ◯ R5000-R9999
- ◯ R10000+

HE10. Source

- ◯ Wages/Income
- ◯ Pension
- ◯ Disability grant
- ◯ Child grant
- ◯ Other

HE10.1 Describe other

____

Household member - 08

HE08. Occupation status

- ◯ Formal employ (full-time)
- ◯ Formal employ (part-time)
- ◯ Self-employed
- ◯ Informal
- ◯ Piece jobs
- ◯ Student - FET/Tertiary
- ◯ In-school learner
- ◯ Pension
- ◯ Unemployed - Looking for work
- ◯ Unemployed - Not looking for work
- ◯ Pre-school going
- ◯ Other

HE08.1 Describe other

____

HE09. Amount earned per month

- ◯ R0
- ◯ R1-R999
- ◯ R1000-R4999
- ◯ R5000-R9999
- ◯ R10000+

HE10. Source	◯ Wages/Income<br>◯ Pension<br>◯ Disability grant<br>◯ Child grant<br>◯ Other
HE10.1 Describe other	__________________________________

Household member - 09

HE08. Occupation status	◯ Formal employ (full-time)<br>◯ Formal employ (part-time)<br>◯ Self-employed<br>◯ Informal<br>◯ Piece jobs<br>◯ Student - FET/Tertiary<br>◯ In-school learner<br>◯ Pension<br>◯ Unemployed - Looking for work<br>◯ Unemployed - Not looking for work<br>◯ Pre-school going<br>◯ Other
HE08.1 Describe other	__________________________________
HE09. Amount earned per month	◯ R0<br>◯ R1-R999<br>◯ R1000-R4999<br>◯ R5000-R9999<br>◯ R10000+
HE10. Source	◯ Wages/Income<br>◯ Pension<br>◯ Disability grant<br>◯ Child grant<br>◯ Other
HE10.1 Describe other	__________________________________

Household member - 10

HE08. Occupation status	◯ Formal employ (full-time)<br>◯ Formal employ (part-time)<br>◯ Self-employed<br>◯ Informal<br>◯ Piece jobs<br>◯ Student - FET/Tertiary<br>◯ In-school learner<br>◯ Pension<br>◯ Unemployed - Looking for work<br>◯ Unemployed - Not looking for work<br>◯ Pre-school going<br>◯ Other
HE08.1 Describe other	__________________________________

HE09. Amount earned per month

◯ R0
◯ R1-R999
◯ R1000-R4999
◯ R5000-R9999
◯ R10000+

HE10. Source

◯ Wages/Income
◯ Pension
◯ Disability grant
◯ Child grant
◯ Other

HE10.1 Describe other

____

Health system and household Costs

HE11. Can you tell us if you/they had any other chronic illnesses before - or in addition to - TB for which they are receiving treatment?

◯ HIV
◯ High blood pressure/Hypertension
◯ Diabetes (needing injection)
◯ Diabetes (needing tablets)
◯ Heart problems
◯ Cancer
◯ Other
◯ Not receiving treatment for any other chronic disease

HE11.1. Site of Cancer

____

HE11.2. Describe other

____

HE12. How many visits to the following services were made by the Index case in relation to symptoms of this TB(or TB/HIV) illness in the 6 months before the diagnosis?

☐ 1. Government clinic or health centre
☐ 2. Private GP
☐ 3. Government hospital outpatient
☐ 4. Government hospital casualty
☐ 5. Traditional healer/sangoma/nyanga
☐ 6. Faith healer

HE12.1. Number of visits - 12.1.

____

HE12.1. Number of visits - 12.2.

____

HE12.1. Number of visits - 12.3.

____

HE12.1. Number of visits - 12.4.

____

HE12.1. Number of visits - 12.5.

____

HE12.1. Number of visits - 12.6.

____

HE13. Did the person with TB or the Index case with TB had to be admitted to a hospital in the 6 months before this TB illness?	◯ Yes<br>◯ No
HE14.1. Hospital	__________________________________
HE14.2. Number of nights	__________________________________
HE15. Complaint/Diagnosis	__________________________________
HE16. From-	__________________________________
HE16. To-	__________________________________
Add another?	◯ Yes<br>◯ No
HE17.1. Hospital	__________________________________
HE17.2. Number of nights	__________________________________
HE18. Complaint/Diagnosis	__________________________________
HE19. From-	__________________________________
HE19. To-	__________________________________
Add another?	◯ Yes<br>◯ No
HE20.1. Hospital	__________________________________
HE20.2. Number of nights	__________________________________
HE21. Complaint/Diagnosis	__________________________________
HE22. From-	__________________________________
HE22. To-	__________________________________

HE23. How much did you pay for the services for each visit that the Index Case used?

- [ ] 1. Government clinic or health centre
- [ ] 2. Private GP
- [ ] 3. Government hospital outpatient
- [ ] 4. Government hospital casualty
- [ ] 5. Private hospital casualty
- [ ] 6. Traditional healer/sangoma/nyanga
- [ ] 7. Faith healer

HE24. ________ZAR

____
(ZAR. (0 if no cost. 9999 if unknown))

HE25. ________ZAR

____
(ZAR. (0 if no cost. 9999 if unknown))

HE26. ________ZAR

____
(ZAR. (0 if no cost. 9999 if unknown))

HE27. ________ZAR

____
(ZAR. (0 if no cost. 9999 if unknown))

HE28. ________ZAR

____
(ZAR. (0 if no cost. 9999 if unknown))

HE29. ________ZAR

____
(ZAR. (0 if no cost. 9999 if unknown))

HE30. ________ZAR

____
(ZAR. (0 if no cost. 9999 if unknown))

HE31. Do you have any kind of medical aid or other assistance with paying the health care costs?

- ◯ Yes - in above amount
- ◯ Yes - on top of above
- ◯ No

HE32. Medical Aid name

____

HE33. Medical Aid cost

____
(ZAR. (0 if no cost. 9999 if unknown))

HE34. Employer help

____
(ZAR. (0 if no cost. 9999 if unknown))

HE35. Other

____
(ZAR. (0 if no cost. 9999 if unknown))

HE36. Has index case/or you taken off from work because of this illness?	◯ Yes<br>◯ No<br>◯ Does not work
HE37. How many working days	__________________________________
HE38. Has index case/you lost income because of this illness?	◯ Yes<br>◯ No<br>◯ Does not have an income
HE39.	__________________________________<br>(ZAR. (0 if no cost. 9999 if unknown))
HE40. How does the index case/you usually travel to the hospital to get care?	☐ Private car<br>☐ Minibus or other taxi<br>☐ Bus<br>☐ Train<br>☐ Walk<br>☐ Other
HE40. Other	__________________________________
HE41. What is the cost of one trip to get care	__________________________________<br>(ZAR. (0 if no cost. 9999 if unknown))
HE42. How far does the index case have to travel to get care?	__________________________________<br>(km)
HE43. How much does the index spend on special treatments, supplements, vitamins or foods because of their sickness?	
HE43a. Fruit and Vegetables	◯ Yes ◯ No
HE43b. Milk	◯ Yes ◯ No
HE43c. Other drinks	◯ Yes ◯ No
HE43d. Vitamins/Herbs	◯ Yes ◯ No
HE43e. Meat	◯ Yes ◯ No
HE43f. Eggs	◯ Yes ◯ No
HE43g. Traditional Medicines	◯ Yes ◯ No
HE43h. Other	◯ Yes ◯ No
Specify	__________________________________

HE44. Overall amount spent on special treatments, supplements, vitamins or foods per month	______________ (ZAR)
HE45. Did anyone go with the index/you to the clinic or hospital?	◯ Yes ◯ No
HE46. How many adults	______________
HE46. Under 18 yrs	______________
HE47. How many times did that person/those people go with the index to the clinic or hospital?	______________ (times)
HE48. How much does the person(s) who accompanied the index earn per day?	
HE49. Person 1	______________ (ZAR. (0 if no cost. 9999 if unknown))
HE50. Person 2	______________ (ZAR. (0 if no cost. 9999 if unknown))
HE51. Person 3	______________ (ZAR. (0 if no cost. 9999 if unknown))
HE52. Why did the index need to be accompanied to the clinic or hospital?	☐ Friendship/comfort/love/emotional support<br>☐ Security<br>☐ Index too sick to manage alone (short of breath, weak etc.)<br>☐ Index mentally incapable of managing alone<br>☐ Speak to health staff to explain condition & understand treatment<br>☐ Other (specify)<br>☐ Mostly went alone
HE52. Other	______________
HE53a. If index was admitted to hospital, how many people visited?	______________
HE53b. Number of visits	______________
HE53a. If index was admitted to hospital, how many people visited?	______________
HE53b. Number of visits	______________

HE53a. If index was admitted to hospital, how many people visited?	______________
HE53b. Number of visits	______________

Coping costs

HE54. Did the person with TB or someone in the household use their own money in the bank, or own money in a stokvel or elsewhere to pay for costs or expenses related to their TB?	◯ Yes<br>◯ No<br>◯ Don't know
HE55. How much	______________<br>(ZAR. (0 if no cost. 9999 if unknown))
HE56. Did the index case/family member borrow money for care or cover costs due to the illness ? (separate from own money in QHE54)	◯ Yes<br>◯ No<br>◯ Don't know
HE57. How much	______________<br>(ZAR)
HE58. When was the money borrowed?	______________
HE58. or,	☐ Don't know
HE59. From whom did you/they borrow money from?	☐ Family<br>☐ Neighbours/Friend<br>☐ Bank<br>☐ Stokvel<br>☐ Machonisa/loan shark<br>☐ Employer<br>☐ Didn't borrow<br>☐ Other
HE59. Other	______________
HE60. How much will you have to pay back and when?	
Amount 1	☐
Specify amount 1	______________<br>(ZAR. (0 if no cost. 9999 if unknown))
Amount 1, after how long	______________
Amount 1: after how long	◯ weeks<br>◯ months

Amount 2	☐
Specify amount 2	__________ (ZAR. (0 if no cost. 9999 if unknown))
Amount 2, after how long	__________
Amount 2: after how long	◯ weeks<br>◯ months
Amount 3	☐
Specify amount 3	__________ (ZAR. (0 if no cost. 9999 if unknown))
Amount 3, after how long	__________
Amount 3: after how long	◯ weeks<br>◯ months
Interest	☐
Specify interest rate	__________ (%)
Interest over how long	__________
Interest over how long	◯ weeks<br>◯ months
I am not expected to pay back the money	☐
Didn't borrow	☐
HE61. Have you paid the money back in full?	◯ Yes paid in full<br>◯ Paid partly<br>◯ Only paid the interest<br>◯ Didn't borrow
HE62. Did the index case/family member have to sell an asset/livestock to get care?	◯ Yes ◯ No
HE63. What asset/s or livestock?	__________
HE64. Total value of all asset/s or livestock sold:	__________ (ZAR. (0 if no cost. 9999 if unknown))

HE65. How much did you actually get when you sold?

______________________ (ZAR)

HE66. Has the household had to reduce spending elsewhere?

○ Yes ○ No

HE67. What did the household reduce spending on the most?

☐ 1. Food
☐ 2. Petrol
☐ 3. Clothing
☐ 4. Airtime
☐ 5. Entertainment
☐ 6. School expenditure
☐ 7. Alcohol
☐ 8. Other

HE67. Other

____

HE68. What other things did they reduce spending on?

☐ 1. Food
☐ 2. Petrol
☐ 3. Clothing
☐ 4. Airtime
☐ 5. Entertainment
☐ 6. School expenditure
☐ 7. Alcohol
☐ 8. Other

HE69. Are other people involved with the care of the index case?

○ Yes ○ No

HE70. Did these people/person have to stop working to care for the index case?

○ Yes ○ No

HE70a Person 1

______________________ (weeks)

HE70b Person 2

______________________ (weeks)

HE70c Person 3

______________________ (weeks)

HE71. What is the monthly income earned by the people/person that looks after the index case?

HE71a: Person 1

○ R0
○ R1-R999
○ R1000-R4999
○ R5000-R9999
○ +R10000

HE71b: Person 2	◯ R0<br>◯ R1-R999<br>◯ R1000-R4999<br>◯ R5000-R9999<br>◯ +R10000
HE71c: Person 3	◯ R0<br>◯ R1-R999<br>◯ R1000-R4999<br>◯ R5000-R9999<br>◯ +R10000
HE72. Did any person lose or leave their job permanently to take of the index case?	◯ Yes ◯ No
HE72a: Person 1	◯ R0<br>◯ R1-R999<br>◯ R1000-R4999<br>◯ R5000-R9999<br>◯ +R10000
HE72b: Person 2	◯ R0<br>◯ R1-R999<br>◯ R1000-R4999<br>◯ R5000-R9999<br>◯ +R10000
HE72c: Person 3	◯ R0<br>◯ R1-R999<br>◯ R1000-R4999<br>◯ R5000-R9999<br>◯ +R10000

Other illness and Additional cost of TB illness

HE73. have there been any additional costs or health service visits for the index because of the other chronic illnesses mentioned above	◯ Yes ◯ No
HE74. What are the other costs?	☐ Clinic visits (per 6 months)<br>☐ Hospital outpatient visits (per 6 months)<br>☐ Hospital admission (nights in last 6 months)<br>☐ Tests or drugs<br>☐ Transport or food<br>☐ Other
HE74. Tests or drugs, cost:	____________________<br>(ZAR. (0 if no cost. 9999 if unknown))
HE74. Transport or food, cost:	____________________<br>(ZAR. (0 if no cost. 9999 if unknown))
HE74. Other	____________________

HE75. On average, how much did the index spend per month on health care costs before the TB illness?

____
(ZAR. (0 if no cost. 9999 if unknown))

HE76. How much does the index spend per month towards health care costs now after the TB illness?

____
(ZAR. (0 if no cost. 9999 if unknown))

If the index case has died

HE77. How much did funeral arrangements cost your household?

____
(ZAR)

HE78. How much was paid with your household money?

____
(ZAR)

HE79. How much was paid by guests, friends or family?

____
(ZAR)

HE80. How much was paid by a burial society or funeral insurance?

____
(ZAR)

HE81. How much was borrowed by your household?

____
(ZAR)

HE82 If loan(s) has to be repaid, how much and by when?

○ How much ○ Interest rate

HE82. How much

____
(ZAR)

HE82. What interest rate

____
(%)

HE83. By when:

____

HE84. How much do you still owe?

____
(ZAR)

HE85. How many livestock did the household contribute to the funeral?
(Do not include any that were bought and included in payments under HE78, 79 or 80)

☐ Cow
☐ Goat
☐ Chicken
☐ Sheep
☐ Pigs
☐ Not applicable

HE85.1. Number of cows

____

HE85.2. Number of goats

____

HE85.3. Number of chicken

____

HE85.4. Number of sheep

____

HE85.5. Number of pigs

____

Overall impact

HE87. On a scale of 1 to 5, in which 1 is no impact and 5 is very serious impact, to what extent has the TB illness affected the family costs and income?

◯ 1-No impact
◯ 2-Little impact
◯ 3-Moderate impact
◯ 4-Serious impact
◯ 5-Very serious impact

HE88. Have any household members been sent to other households because yours could not cope since the index illness started?

◯ Yes ◯ No

HE89. How many members?

____

HE90. Have any learners/ students in the household dropped out from learning since the index illness started?

◯ Yes ◯ No

Dropped for more than 3 months

☐

HE90. No.

____

Dropped for more than 3 months

☐

HE90. No.

____

Quality of Life

HE91. What do you expect will happen to the quality of life for the people in your household over the next year?

◯ Much better than 1 year ago
◯ Somewhat better than 1 year ago
◯ About the same
◯ Somewhat worse than one year ago
◯ Much worse than one year ago

HE92. In general the health of the index case is?

◯ Excellent
◯ Very good
◯ Good
◯ Fair
◯ Poor

HE93. Compared to one year ago the index is?	◯ Much better than 1 year ago<br>◯ Somewhat better than 1 year ago<br>◯ About the same<br>◯ Somewhat worse than one year ago<br>◯ Much worse than one year ago
HE94. Mobility	◯ I have no problems in walking about<br>◯ I have slight problems in walking about<br>◯ I have moderate problems in walking about<br>◯ I have severe problems in walking about<br>◯ I am unable to walk about
HE95. Self Care	◯ I have no problems washing or dressing myself<br>◯ I have slight problems washing or dressing myself<br>◯ I have moderate problems washing or dressing myself<br>◯ I have severe problems washing or dressing myself<br>◯ I am unable to wash or dress myself
HE96. Usual activities	◯ I have no problems doing my usual activities<br>◯ I have slight problems doing my usual activities<br>◯ I have moderate problems doing my usual activities<br>◯ I have severe problems doing my usual activities<br>◯ I am unable to do my usual activities
HE97. Pain/discomfort	◯ I have no pain or discomfort<br>◯ I have slight pain or discomfort<br>◯ I have moderate pain or discomfort<br>◯ I have severe pain or discomfort<br>◯ I have extreme pain or discomfort
HE98. Anxiety/depression	◯ I am not anxious or depressed<br>◯ I am slightly anxious or depressed<br>◯ I am moderately anxious or depressed<br>◯ I am severely anxious or depressed<br>◯ I am extremely anxious or depressed
HE93. In the past two weeks	
HE93.1 Does the index case have enough energy for everyday life?	☐
HE93.1 Score	◯ 1<br>◯ 2<br>◯ 3<br>◯ 4<br>◯ 5
HE93.2 Does the index case have enough money to meet his/her needs?	☐
HE93.2 Score	◯ 1<br>◯ 2<br>◯ 3<br>◯ 4<br>◯ 5
HE93.3 Does the index case feel accepted by the people that he/she knows?	☐

HE93.3 Score	◯ 1<br>◯ 2<br>◯ 3<br>◯ 4<br>◯ 5
HE93.4 Does the index case feel life is meaningful?	☐
HE93.4 Score	◯ 1<br>◯ 2<br>◯ 3<br>◯ 4<br>◯ 5
HE93.5 How much does the index case enjoy life?	☐
HE93.5 Score	◯ 1<br>◯ 2<br>◯ 3<br>◯ 4<br>◯ 5
HE100. How satisfied is the index case with his/her quality of sleep?	◯ Very good<br>◯ Good<br>◯ Neither good nor bad<br>◯ Poor<br>◯ Very poor
HE101. To what extent is the index case troubled by people knowing that he/she has TB ?<br><br>(rate out of 5, with 5 being very troubled and 1 least troubled)<br>(If a child: to what extent will it create trouble for the child if people know that he/she has TB?)	◯ 1<br>◯ 2<br>◯ 3<br>◯ 4<br>◯ 5
HE102. To what extent is the index case troubled by people knowing that he/she has HIV? [if relevant]<br><br>(rate out of 5, with 5 being very troubled and 1 least troubled)<br>(If a child: to what extent will it create trouble for the child if people know that he/she has TB?)	◯ 1<br>◯ 2<br>◯ 3<br>◯ 4<br>◯ 5

Form Completion

HE103. Date form HE completed	______________________________
HE104. Name of person completing this form	______________________________

Audit Trail

Name of Data Entry Person

____

Date of Data Entry

____
(Kindly change the date every time a change is made to the data)

Form Version: 1.1 dated 14 June 2017

____

Health Economics Form HE_M15

Form completed at:	◯ Month 15
i. Start Time	__________________

Baseline

ii. Date of Interview	__________________
iii. Status of Index Case	◯ Alive<br>◯ Deceased
iii.i. Date decesaed	__________________
iv. Date of TB diagnosis	__________________
v. Who is the main person providing information for this questionnaire?	◯ Index case<br>◯ Other
v.i. Relation to Index case	◯ Wife/Mother<br>◯ Husband/Father<br>◯ Extended family<br>◯ Son/Daughter<br>◯ Other
v.i.i. Describe other	__________________

Household Socio-economic Status

HE01. How many people in the household receive an income?<br>(including pensions and grants)	__________________
HE02. Who is the primary income earner in the household?	◯ Index<br>◯ Wife/Mother<br>◯ Husband/Father<br>◯ Extended family<br>◯ Son/Daughter<br>◯ Other
HE02.1 Describe other	__________________

HE03. Does your household have any of the following and how many?

- ☐ Refrigerator
- ☐ Stove
- ☐ Television
- ☐ Car
- ☐ Radio
- ☐ Phone-landline
- ☐ Internet
- ☐ Microwave
- ☐ Cellphone
- ☐ Smartphone
- ☐ Computer
- ☐ Washing machine
- ☐ Satellite dish/DSTV

HE03.01. Number - Refrigirator ________________________

HE03.02. Number - Stove ________________________

HE03.03. Number - Television ________________________

HE03.04. Number - Car ________________________

HE03.05. Number - Radio ________________________

HE03.06. Number - Phone-landline ________________________

HE03.07. Number - Internet ________________________

HE03.08. Number - Microwave ________________________

HE03.09. Number - Cellphone ________________________

HE03.10. Number - Smartphone ________________________

HE03.11. Number - Computer ________________________

HE03.12. Number - Washing machine ________________________

HE03.13. Number - Satellite dish/DSTV ________________________

HE04. Household food production

- ☐ Livestock
- ☐ Mielies/Sorghum
- ☐ Vegetables
- ☐ Poultry
- ☐ Other

HE04.1. Describe other

____

HE05. Do you have additional income sent by household members not living with you?

- ◯ Yes
- ◯ No

HE05.1. How many?

____

HE06. Amount.R-

____

HE07. Household members

____

Household member - 01

HE08. Occupation status

- ◯ Formal employ (full-time)
- ◯ Formal employ (part-time)
- ◯ Self-employed
- ◯ Informal
- ◯ Piece jobs
- ◯ Student - FET/Tertiary
- ◯ In-school learner
- ◯ Pension
- ◯ Unemployed - Looking for work
- ◯ Unemployed - Not looking for work
- ◯ Pre-school going
- ◯ Other

HE08.1 Describe other

____

HE09. Amount earned per month

- ◯ R0
- ◯ R1-R999
- ◯ R1000-R4999
- ◯ R5000-R9999
- ◯ R10000+

HE10. Source

- ◯ Wages/Income
- ◯ Pension
- ◯ Disability grant
- ◯ Child grant
- ◯ Other

HE10.1 Describe other

____

Household member - 02

HE08. Occupation status
- ◯ Formal employ (full-time)
- ◯ Formal employ (part-time)
- ◯ Self-employed
- ◯ Informal
- ◯ Piece jobs
- ◯ Student - FET/Tertiary
- ◯ In-school learner
- ◯ Pension
- ◯ Unemployed - Looking for work
- ◯ Unemployed - Not looking for work
- ◯ Pre-school going
- ◯ Other

HE08.1 Describe other ______________________________

HE09. Amount earned per month
- ◯ R0
- ◯ R1-R999
- ◯ R1000-R4999
- ◯ R5000-R9999
- ◯ R10000+

HE10. Source
- ◯ Wages/Income
- ◯ Pension
- ◯ Disability grant
- ◯ Child grant
- ◯ Other

HE10.1 Describe other ______________________________

Household member - 03

HE08. Occupation status
- ◯ Formal employ (full-time)
- ◯ Formal employ (part-time)
- ◯ Self-employed
- ◯ Informal
- ◯ Piece jobs
- ◯ Student - FET/Tertiary
- ◯ In-school learner
- ◯ Pension
- ◯ Unemployed - Looking for work
- ◯ Unemployed - Not looking for work
- ◯ Pre-school going
- ◯ Other

HE08.1 Describe other ______________________________

HE09. Amount earned per month
- ◯ R0
- ◯ R1-R999
- ◯ R1000-R4999
- ◯ R5000-R9999
- ◯ R10000+

HE10. Source

◯ Wages/Income
◯ Pension
◯ Disability grant
◯ Child grant
◯ Other

HE10.1 Describe other

____

Household member - 04

HE08. Occupation status

◯ Formal employ (full-time)
◯ Formal employ (part-time)
◯ Self-employed
◯ Informal
◯ Piece jobs
◯ Student - FET/Tertiary
◯ In-school learner
◯ Pension
◯ Unemployed - Looking for work
◯ Unemployed - Not looking for work
◯ Pre-school going
◯ Other

HE08.1 Describe other

____

HE09. Amount earned per month

◯ R0
◯ R1-R999
◯ R1000-R4999
◯ R5000-R9999
◯ R10000+

HE10. Source

◯ Wages/Income
◯ Pension
◯ Disability grant
◯ Child grant
◯ Other

HE10.1 Describe other

____

Household member - 05

HE08. Occupation status

◯ Formal employ (full-time)
◯ Formal employ (part-time)
◯ Self-employed
◯ Informal
◯ Piece jobs
◯ Student - FET/Tertiary
◯ In-school learner
◯ Pension
◯ Unemployed - Looking for work
◯ Unemployed - Not looking for work
◯ Pre-school going
◯ Other

HE08.1 Describe other

____

HE09. Amount earned per month	◯ R0<br>◯ R1-R999<br>◯ R1000-R4999<br>◯ R5000-R9999<br>◯ R10000+
HE10. Source	◯ Wages/Income<br>◯ Pension<br>◯ Disability grant<br>◯ Child grant<br>◯ Other
HE10.1 Describe other	__________________________________

Household member - 06

HE08. Occupation status	◯ Formal employ (full-time)<br>◯ Formal employ (part-time)<br>◯ Self-employed<br>◯ Informal<br>◯ Piece jobs<br>◯ Student - FET/Tertiary<br>◯ In-school learner<br>◯ Pension<br>◯ Unemployed - Looking for work<br>◯ Unemployed - Not looking for work<br>◯ Pre-school going<br>◯ Other
HE08.1 Describe other	__________________________________
HE09. Amount earned per month	◯ R0<br>◯ R1-R999<br>◯ R1000-R4999<br>◯ R5000-R9999<br>◯ R10000+
HE10. Source	◯ Wages/Income<br>◯ Pension<br>◯ Disability grant<br>◯ Child grant<br>◯ Other
HE10.1 Describe other	__________________________________

Household member - 07

HE08. Occupation status

- ◯ Formal employ (full-time)
- ◯ Formal employ (part-time)
- ◯ Self-employed
- ◯ Informal
- ◯ Piece jobs
- ◯ Student - FET/Tertiary
- ◯ In-school learner
- ◯ Pension
- ◯ Unemployed - Looking for work
- ◯ Unemployed - Not looking for work
- ◯ Pre-school going
- ◯ Other

HE08.1 Describe other

____

HE09. Amount earned per month

- ◯ R0
- ◯ R1-R999
- ◯ R1000-R4999
- ◯ R5000-R9999
- ◯ R10000+

HE10. Source

- ◯ Wages/Income
- ◯ Pension
- ◯ Disability grant
- ◯ Child grant
- ◯ Other

HE10.1 Describe other

____

Household member - 08

HE08. Occupation status

- ◯ Formal employ (full-time)
- ◯ Formal employ (part-time)
- ◯ Self-employed
- ◯ Informal
- ◯ Piece jobs
- ◯ Student - FET/Tertiary
- ◯ In-school learner
- ◯ Pension
- ◯ Unemployed - Looking for work
- ◯ Unemployed - Not looking for work
- ◯ Pre-school going
- ◯ Other

HE08.1 Describe other

____

HE09. Amount earned per month

- ◯ R0
- ◯ R1-R999
- ◯ R1000-R4999
- ◯ R5000-R9999
- ◯ R10000+

HE10. Source	◯ Wages/Income<br>◯ Pension<br>◯ Disability grant<br>◯ Child grant<br>◯ Other
HE10.1 Describe other	__________________________________

Household member - 09

HE08. Occupation status	◯ Formal employ (full-time)<br>◯ Formal employ (part-time)<br>◯ Self-employed<br>◯ Informal<br>◯ Piece jobs<br>◯ Student - FET/Tertiary<br>◯ In-school learner<br>◯ Pension<br>◯ Unemployed - Looking for work<br>◯ Unemployed - Not looking for work<br>◯ Pre-school going<br>◯ Other
HE08.1 Describe other	__________________________________
HE09. Amount earned per month	◯ R0<br>◯ R1-R999<br>◯ R1000-R4999<br>◯ R5000-R9999<br>◯ R10000+
HE10. Source	◯ Wages/Income<br>◯ Pension<br>◯ Disability grant<br>◯ Child grant<br>◯ Other
HE10.1 Describe other	__________________________________

Household member - 10

HE08. Occupation status	◯ Formal employ (full-time)<br>◯ Formal employ (part-time)<br>◯ Self-employed<br>◯ Informal<br>◯ Piece jobs<br>◯ Student - FET/Tertiary<br>◯ In-school learner<br>◯ Pension<br>◯ Unemployed - Looking for work<br>◯ Unemployed - Not looking for work<br>◯ Pre-school going<br>◯ Other
HE08.1 Describe other	__________________________________

HE09. Amount earned per month	◯ R0<br>◯ R1-R999<br>◯ R1000-R4999<br>◯ R5000-R9999<br>◯ R10000+
HE10. Source	◯ Wages/Income<br>◯ Pension<br>◯ Disability grant<br>◯ Child grant<br>◯ Other
HE10.1 Describe other	__________________________________

Health system and Household Costs

HE11. Can you tell us if you/they had any other chronic illnesses before - or in addition to - TB for which they are receiving treatment?	◯ HIV<br>◯ High blood pressure/Hypertension<br>◯ Diabetes (needing injection)<br>◯ Diabetes (needing tablets)<br>◯ Heart problems<br>◯ Cancer<br>◯ Other<br>◯ Not receiving treatment for any other chronic disease
HE11.1. Site of Cancer	__________________________________
HE11.2. Describe other	__________________________________
HE12. How many visits to the following services were made by the Index case in relation to symptoms of this TB(or TB/HIV) illness in the 6 months before the diagnosis?	☐ 1. Government clinic or health centre<br>☐ 2. Private GP<br>☐ 3. Government hospital outpatient<br>☐ 4. Government hospital casualty<br>☐ 5. Traditional healer/sangoma/nyanga<br>☐ 6. Faith healer
HE12.1. Number of visits - 12.1.	__________________________________
HE12.1. Number of visits - 12.2.	__________________________________
HE12.1. Number of visits - 12.3.	__________________________________
HE12.1. Number of visits - 12.4.	__________________________________
HE12.1. Number of visits - 12.5.	__________________________________
HE12.1. Number of visits - 12.6.	__________________________________

HE13. Did the person with TB or the Index case with TB had to be admitted to a hospital in the 6 months before this TB illness?	◯ Yes<br>◯ No
HE14.1. Hospital	__________________________________
HE14.2. Number of nights	__________________________________
HE15. Complaint/Diagnosis	__________________________________
HE16. From-	__________________________________
HE16. To-	__________________________________
Add another?	◯ Yes<br>◯ No
HE17.1. Hospital	__________________________________
HE17.2. Number of nights	__________________________________
HE18. Complaint/Diagnosis	__________________________________
HE19. From-	__________________________________
HE19. To-	__________________________________
Add another?	◯ Yes<br>◯ No
HE20.1. Hospital	__________________________________
HE20.2. Number of nights	__________________________________
HE21. Complaint/Diagnosis	__________________________________
HE22. From-	__________________________________
HE22. To-	__________________________________

HE23. How much did you pay for the services for each visit that the Index Case used?

- ☐ 1. Government clinic or health centre
- ☐ 2. Private GP
- ☐ 3. Government hospital outpatient
- ☐ 4. Government hospital casualty
- ☐ 5. Private hospital casualty
- ☐ 6. Traditional healer/sangoma/nyanga
- ☐ 7. Faith healer

HE24. ________ZAR

____
(ZAR. (0 if no cost. 9999 if unknown))

HE25. ________ZAR

____
(ZAR. (0 if no cost. 9999 if unknown))

HE26. ________ZAR

____
(ZAR. (0 if no cost. 9999 if unknown))

HE27. ________ZAR

____
(ZAR. (0 if no cost. 9999 if unknown))

HE28. ________ZAR

____
(ZAR. (0 if no cost. 9999 if unknown))

HE29. ________ZAR

____
(ZAR. (0 if no cost. 9999 if unknown))

HE30. ________ZAR

____
(ZAR. (0 if no cost. 9999 if unknown))

HE31. Do you have any kind of medical aid or other assistance with paying the health care costs?

- ◯ Yes - in above amount
- ◯ Yes - on top of above
- ◯ No

HE32. Medical Aid name

____

HE33. Medical Aid cost

____
(ZAR. (0 if no cost. 9999 if unknown))

HE34. Employer help

____
(ZAR. (0 if no cost. 9999 if unknown))

HE35. Other

____
(ZAR. (0 if no cost. 9999 if unknown))

HE36. Has index case/or you taken off from work because of this illness?	◯ Yes<br>◯ No<br>◯ Does not work
HE37. How many working days	__________________________________
HE38. Has index case/you lost income because of this illness?	◯ Yes<br>◯ No<br>◯ Does not have an income
HE39.	__________________________________<br>(ZAR. (0 if no cost. 9999 if unknown))
HE40. How does the index case/you usually travel to the hospital to get care?	☐ Private car<br>☐ Minibus or other taxi<br>☐ Bus<br>☐ Train<br>☐ Walk<br>☐ Other
HE40. Other	__________________________________
HE41. What is the cost of one trip to get care	__________________________________<br>(ZAR. (0 if no cost. 9999 if unknown))
HE42. How far does the index case have to travel to get care?	__________________________________<br>(km)
HE43. How much does the index spend on special treatments, supplements, vitamins or foods because of their sickness?	
HE43a. Fruit and Vegetables	◯ Yes ◯ No
HE43b. Milk	◯ Yes ◯ No
HE43c. Other drinks	◯ Yes ◯ No
HE43d. Vitamins/Herbs	◯ Yes ◯ No
HE43e. Meat	◯ Yes ◯ No
HE43f. Eggs	◯ Yes ◯ No
HE43g. Traditional Medicines	◯ Yes ◯ No
HE43h. Other	◯ Yes ◯ No
Specify	__________________________________

HE44. Overall amount spent on special treatments, supplements, vitamins or foods per month	__________ (ZAR)
HE45. Did anyone go with the index/you to the clinic or hospital?	◯ Yes ◯ No
HE46. How many adults	__________
HE46. Under 18 yrs	__________
HE47. How many times did that person/those people go with the index to the clinic or hospital?	__________ (times)
HE48. How much does the person(s) who accompanied the index earn per day?	
HE49. Person 1	__________ (ZAR. (0 if no cost. 9999 if unknown))
HE50. Person 2	__________ (ZAR. (0 if no cost. 9999 if unknown))
HE51. Person 3	__________ (ZAR. (0 if no cost. 9999 if unknown))
HE52. Why did the index need to be accompanied to the clinic or hospital?	☐ Friendship/comfort/love/emotional support<br>☐ Security<br>☐ Index too sick to manage alone (short of breath, weak etc.)<br>☐ Index mentally incapable of managing alone<br>☐ Speak to health staff to explain condition & understand treatment<br>☐ Other (specify)<br>☐ Mostly went alone
HE52. Other	__________
HE53a. If index was admitted to hospital, how many people visited?	__________
HE53b. Number of visits	__________
HE53a. If index was admitted to hospital, how many people visited?	__________
HE53b. Number of visits	__________

HE53a. If index was admitted to hospital, how many people visited?

____

HE53b. Number of visits

____

Coping costs

HE54. Did the person with TB or someone in the household use their own money in the bank, or own money in a stokvel or elsewhere to pay for costs or expenses related to their TB?

- ◯ Yes
- ◯ No
- ◯ Don't know

HE55. How much

____
(ZAR. (0 if no cost. 9999 if unknown))

HE56. Did the index case/family member borrow money for care or cover costs due to the illness ? (separate from own money in QHE54)

- ◯ Yes
- ◯ No
- ◯ Don't know

HE57. How much

____
(ZAR)

HE58. When was the money borrowed?

____

HE58. or,

- ☐ Don't know

HE59. From whom did you/they borrow money from?

- ☐ Family
- ☐ Neighbours/Friend
- ☐ Bank
- ☐ Stokvel
- ☐ Machonisa/loan shark
- ☐ Employer
- ☐ Didn't borrow
- ☐ Other

HE59. Other

____

HE60. How much will you have to pay back and when?

Amount 1

- ☐

Specify amount 1

____
(ZAR. (0 if no cost. 9999 if unknown))

Amount 1, after how long

____

Amount 1: after how long

- ◯ weeks
- ◯ months

Amount 2	☐
Specify amount 2	____________________ (ZAR. (0 if no cost. 9999 if unknown))
Amount 2, after how long	____________________
Amount 2: after how long	◯ weeks<br>◯ months
Amount 3	☐
Specify amount 3	____________________ (ZAR. (0 if no cost. 9999 if unknown))
Amount 3, after how long	____________________
Amount 3: after how long	◯ weeks<br>◯ months
Interest	☐
Specify interest rate	____________________ (%)
Interest over how long	____________________
Interest over how long	◯ weeks<br>◯ months
I am not expected to pay back the money	☐
Didn't borrow	☐
HE61. Have you paid the money back in full?	◯ Yes paid in full<br>◯ Paid partly<br>◯ Only paid the interest<br>◯ Didn't borrow
HE62. Did the index case/family member have to sell an asset/livestock to get care?	◯ Yes ◯ No
HE63. What asset/s or livestock?	____________________
HE64. Total value of all asset/s or livestock sold:	____________________ (ZAR. (0 if no cost. 9999 if unknown))

HE65. How much did you actually get when you sold?

______________________ (ZAR)

HE66. Has the household had to reduce spending elsewhere?

◯ Yes ◯ No

HE67. What did the household reduce spending on the most?

☐ 1. Food
☐ 2. Petrol
☐ 3. Clothing
☐ 4. Airtime
☐ 5. Entertainment
☐ 6. School expenditure
☐ 7. Alcohol
☐ 8. Other

HE67. Other

____

HE68. What other things did they reduce spending on?

☐ 1. Food
☐ 2. Petrol
☐ 3. Clothing
☐ 4. Airtime
☐ 5. Entertainment
☐ 6. School expenditure
☐ 7. Alcohol
☐ 8. Other

HE69. Are other people involved with the care of the index case?

◯ Yes ◯ No

HE70. Did these people/person have to stop working to care for the index case?

◯ Yes ◯ No

HE70a Person 1

______________________ (weeks)

HE70b Person 2

______________________ (weeks)

HE70c Person 3

______________________ (weeks)

HE71. What is the monthly income earned by the people/person that looks after the index case?

HE71a: Person 1

◯ R0
◯ R1-R999
◯ R1000-R4999
◯ R5000-R9999
◯ +R10000

HE71b: Person 2	◯ R0<br>◯ R1-R999<br>◯ R1000-R4999<br>◯ R5000-R9999<br>◯ +R10000
HE71c: Person 3	◯ R0<br>◯ R1-R999<br>◯ R1000-R4999<br>◯ R5000-R9999<br>◯ +R10000
HE72. Did any person lose or leave their job permanently to take of the index case?	◯ Yes ◯ No
HE72a: Person 1	◯ R0<br>◯ R1-R999<br>◯ R1000-R4999<br>◯ R5000-R9999<br>◯ +R10000
HE72b: Person 2	◯ R0<br>◯ R1-R999<br>◯ R1000-R4999<br>◯ R5000-R9999<br>◯ +R10000
HE72c: Person 3	◯ R0<br>◯ R1-R999<br>◯ R1000-R4999<br>◯ R5000-R9999<br>◯ +R10000

Other illness and Additional cost of TB illness

HE73. have there been any additional costs or health service visits for the index because of the other chronic illnesses mentioned above	◯ Yes ◯ No
HE74. What are the other costs?	☐ Clinic visits (per 6 months)<br>☐ Hospital outpatient visits (per 6 months)<br>☐ Hospital admission (nights in last 6 months)<br>☐ Tests or drugs<br>☐ Transport or food<br>☐ Other
HE74. Tests or drugs, cost:	______________________<br>(ZAR. (0 if no cost. 9999 if unknown))
HE74. Transport or food, cost:	______________________<br>(ZAR. (0 if no cost. 9999 if unknown))
HE74. Other	______________________

HE75. On average, how much did the index spend per month on health care costs before the TB illness?

____
(ZAR. (0 if no cost. 9999 if unknown))

HE76. How much does the index spend per month towards health care costs now after the TB illness?

____
(ZAR. (0 if no cost. 9999 if unknown))

If the index case has died

HE77. How much did funeral arrangements cost your household?

____
(ZAR)

HE78. How much was paid with your household money?

____
(ZAR)

HE79. How much was paid by guests, friends or family?

____
(ZAR)

HE80. How much was paid by a burial society or funeral insurance?

____
(ZAR)

HE81. How much was borrowed by your household?

____
(ZAR)

HE82 If loan(s) has to be repaid, how much and by when?

○ How much ○ Interest rate

HE82. How much

____
(ZAR)

HE82. What interest rate

____
(%)

HE83. By when:

____

HE84. How much do you still owe?

____
(ZAR)

HE85. How many livestock did the household contribute to the funeral?
(Do not include any that were bought and included in payments under HE78, 79 or 80)

- ☐ Cow
- ☐ Goat
- ☐ Chicken
- ☐ Sheep
- ☐ Pigs
- ☐ Not applicable

HE85.1. Number of cows

____

HE85.2. Number of goats __________________________________

HE85.3. Number of chicken __________________________________

HE85.4. Number of sheep __________________________________

HE85.5. Number of pigs __________________________________

Overall impact

HE87. On a scale of 1 to 5, in which 1 is no impact and 5 is very serious impact, to what extent has the TB illness affected the family costs and income?

- ◯ 1-No impact
- ◯ 2-Little impact
- ◯ 3-Moderate impact
- ◯ 4-Serious impact
- ◯ 5-Very serious impact

HE88. Have any household members been sent to other households because yours could not cope since the index illness started?

◯ Yes ◯ No

HE89. How many members? __________________________________

HE90. Have any learners/ students in the household dropped out from learning since the index illness started?

◯ Yes ◯ No

Dropped for more than 3 months ☐

HE90. No. __________________________________

Dropped for more than 3 months ☐

HE90. No. __________________________________

Quality of Life

HE91. What do you expect will happen to the quality of life for the people in your household over the next year?

- ◯ Much better than 1 year ago
- ◯ Somewhat better than 1 year ago
- ◯ About the same
- ◯ Somewhat worse than one year ago
- ◯ Much worse than one year ago

HE92. In general the health of the index case is?

- ◯ Excellent
- ◯ Very good
- ◯ Good
- ◯ Fair
- ◯ Poor

HE93. Compared to one year ago the index is?	◯ Much better than 1 year ago<br>◯ Somewhat better than 1 year ago<br>◯ About the same<br>◯ Somewhat worse than one year ago<br>◯ Much worse than one year ago
HE94. Mobility	◯ I have no problems in walking about<br>◯ I have slight problems in walking about<br>◯ I have moderate problems in walking about<br>◯ I have severe problems in walking about<br>◯ I am unable to walk about
HE95. Self Care	◯ I have no problems washing or dressing myself<br>◯ I have slight problems washing or dressing myself<br>◯ I have moderate problems washing or dressing myself<br>◯ I have severe problems washing or dressing myself<br>◯ I am unable to wash or dress myself
HE96. Usual activities	◯ I have no problems doing my usual activities<br>◯ I have slight problems doing my usual activities<br>◯ I have moderate problems doing my usual activities<br>◯ I have severe problems doing my usual activities<br>◯ I am unable to do my usual activities
HE97. Pain/discomfort	◯ I have no pain or discomfort<br>◯ I have slight pain or discomfort<br>◯ I have moderate pain or discomfort<br>◯ I have severe pain or discomfort<br>◯ I have extreme pain or discomfort
HE98. Anxiety/depression	◯ I am not anxious or depressed<br>◯ I am slightly anxious or depressed<br>◯ I am moderately anxious or depressed<br>◯ I am severely anxious or depressed<br>◯ I am extremely anxious or depressed
HE93. In the past two weeks	
HE93.1 Does the index case have enough energy for everyday life?	☐
HE93.1 Score	◯ 1<br>◯ 2<br>◯ 3<br>◯ 4<br>◯ 5
HE93.2 Does the index case have enough money to meet his/her needs?	☐
HE93.2 Score	◯ 1<br>◯ 2<br>◯ 3<br>◯ 4<br>◯ 5
HE93.3 Does the index case feel accepted by the people that he/she knows?	☐

HE93.3 Score	◯ 1<br>◯ 2<br>◯ 3<br>◯ 4<br>◯ 5
HE93.4 Does the index case feel life is meaningful?	☐
HE93.4 Score	◯ 1<br>◯ 2<br>◯ 3<br>◯ 4<br>◯ 5
HE93.5 How much does the index case enjoy life?	☐
HE93.5 Score	◯ 1<br>◯ 2<br>◯ 3<br>◯ 4<br>◯ 5
HE100. How satisfied is the index case with his/her quality of sleep?	◯ Very good<br>◯ Good<br>◯ Neither good nor bad<br>◯ Poor<br>◯ Very poor
HE101. To what extent is the index case troubled by people knowing that he/she has TB ?<br><br>(rate out of 5, with 5 being very troubled and 1 least troubled)<br>(If a child: to what extent will it create trouble for the child if people know that he/she has TB?)	◯ 1<br>◯ 2<br>◯ 3<br>◯ 4<br>◯ 5
HE102. To what extent is the index case troubled by people knowing that he/she has HIV? [if relevant]<br><br>(rate out of 5, with 5 being very troubled and 1 least troubled)<br>(If a child: to what extent will it create trouble for the child if people know that he/she has TB?)	◯ 1<br>◯ 2<br>◯ 3<br>◯ 4<br>◯ 5

Form Completion

HE103. Date form HE completed	____________________
HE104. Name of person completing this form	____________________

Audit Trail

Name of Data Entry Person

____

Date of Data Entry

____
(Kindly change the date every time a change is made to the data)

Form Version: 1.1 dated 14 June 2017

____

Costing Of Intervention

HH Study ID: __________________________________

Visit Date: __________________________________

Departure time from office/previous household __________________________________

Return time to office/ arrival at next HH __________________________________

Start time of HH visit (on site) __________________________________

Departure time from HH __________________________________

Nearest Clinic/Health Centre to HH (name): __________________________________

Nearest Clinic GPS coordinates __________________________________

Household GPS coordinates __________________________________

Type of visit:
- ◯ 1st
- ◯ Results Feedback
- ◯ Start treatment
- ◯ Other

No one at home so visit cancelled/rescheduled? ◯ Yes ◯ No

Repeat visit due to absent HH member at last visit? ◯ Yes ◯ No

Personnel

Professional nurses

Grade and rank __________________________________

Total Time for visit (Mins) for HH* __________________________________
(* Including preparation, waiting; Travel to and from HH; General introduction, find HH members; Patient records; Informed consent; CRF data collection; Intervention tests etc)

Total time __________________________________

Enrolled nurse	
Grade and rank	______________________
Total Time for visit (Mins) for HH*	______________________<br>(* Including preparation, waiting; Travel to and from HH; General introduction, find HH members; Patient records; Informed consent; CRF data collection; Intervention tests etc)
Total time	______________________
Counsellor	
Grade and rank	______________________
Total Time for visit (Mins) for HH*	______________________<br>(* Including preparation, waiting; Travel to and from HH; General introduction, find HH members; Patient records; Informed consent; CRF data collection; Intervention tests etc)
Total time	______________________
Other (please state)	
Grade and rank	______________________
Total Time for visit (Mins) for HH*	______________________<br>(* Including preparation, waiting; Travel to and from HH; General introduction, find HH members; Patient records; Informed consent; CRF data collection; Intervention tests etc)
Total time	______________________
Other (please state)	
Grade and rank	______________________
Total Time for visit (Mins) for HH*	______________________<br>(* Including preparation, waiting; Travel to and from HH; General introduction, find HH members; Patient records; Informed consent; CRF data collection; Intervention tests etc)

Total time

____

Tests at household

Sputum for Xpert

Number Done

____

Sputum for culture

Number Done

____

HIV test

Number Done

____

TST

Number Done

____

Isoniazid

Number Done

____

Vit B12

Number Done

____

Transport

Vehicle type

- ◯ Car
- ◯ 4x4
- ◯ bakkie
- ◯ minibus
- ◯ other

Distance to HH from nearest clinic/ health centre (not Study Office)

____

Other costs

Calls

Number ______________________

Items ______________________

Cost per item ______________________

Stationary

Number ______________________

Items ______________________

Cost per item ______________________

Consumables

Number ______________________

Items ______________________

Cost per item ______________________

Airtime

Number ______________________

Items ______________________

Cost per item ______________________

Other

Number ______________________

Items ______________________

Cost per item ____________________

Home Visit Log for staff time direct observation of time and motion

Visit Date ____________________

HH Visit Date ____________________

Visit Start time ____________________

Visit End Time ____________________

Number of HH members screened > 15yrs ____________________

Number of HH members screened < 15yrs ____________________

DID THE PROVIDER AGREE TO PARTICIPATE? ◯ Yes ◯ No

Add Obs. 1 ◯ Yes ◯ No

Obs. 1 - Activity Code
◯ A
◯ B
◯ C
◯ D
◯ E
◯ F
◯ G
◯ H
◯ I
◯ J
◯ K
◯ L
◯ M
◯ N
◯ O
◯ P
◯ Q
◯ R
◯ S

Obs. 1 - Start Time ____________________

Obs. 1 - End Time ____________________

Obs. 1 - Notes ____________________

Add Obs. 2	◯ Yes ◯ No
Obs. 2 - Activity Code	◯ A<br>◯ B<br>◯ C<br>◯ D<br>◯ E<br>◯ F<br>◯ G<br>◯ H<br>◯ I<br>◯ J<br>◯ K<br>◯ L<br>◯ M<br>◯ N<br>◯ O<br>◯ P<br>◯ Q<br>◯ R<br>◯ S
Obs. 2 - Start Time	__________________________________
Obs. 2 - End Time	__________________________________
Obs. 2 - Notes	__________________________________
Add Obs. 3	◯ Yes ◯ No
Obs. 3 - Activity Code	◯ A<br>◯ B<br>◯ C<br>◯ D<br>◯ E<br>◯ F<br>◯ G<br>◯ H<br>◯ I<br>◯ J<br>◯ K<br>◯ L<br>◯ M<br>◯ N<br>◯ O<br>◯ P<br>◯ Q<br>◯ R<br>◯ S
Obs. 3 - End Time	__________________________________
Obs. 3 - Start Time	__________________________________

Obs. 3 - Notes
____

Add Obs. 4 ◯ Yes ◯ No

Obs. 4 - Activity Code
◯ A
◯ B
◯ C
◯ D
◯ E
◯ F
◯ G
◯ H
◯ I
◯ J
◯ K
◯ L
◯ M
◯ N
◯ O
◯ P
◯ Q
◯ R
◯ S

Obs. 4 - Start Time
____

Obs. 4 - End Time
____

Obs. 4 - Notes
____

Add Obs. 5 ◯ Yes ◯ No

Obs. 5 - Activity Code
◯ A
◯ B
◯ C
◯ D
◯ E
◯ F
◯ G
◯ H
◯ I
◯ J
◯ K
◯ L
◯ M
◯ N
◯ O
◯ P
◯ Q
◯ R
◯ S

Obs. 5 - Start Time
____

Obs. 5 - End Time

____

Obs. 5 - Notes

____

Add Obs. 6 ◯ Yes ◯ No

Obs. 6 - Activity Code
◯ A
◯ B
◯ C
◯ D
◯ E
◯ F
◯ G
◯ H
◯ I
◯ J
◯ K
◯ L
◯ M
◯ N
◯ O
◯ P
◯ Q
◯ R
◯ S

Obs. 6 - Start Time

____

Obs. 6 - End Time

____

Obs. 6 - Notes

____

Add Obs. 7 ◯ Yes ◯ No

Obs. 7 - Activity Code
◯ A
◯ B
◯ C
◯ D
◯ E
◯ F
◯ G
◯ H
◯ I
◯ J
◯ K
◯ L
◯ M
◯ N
◯ O
◯ P
◯ Q
◯ R
◯ S

Obs. 7 - Start Time	________________
Obs. 7 - End Time	________________
Obs. 7 - Notes	________________
Add Obs. 8	◯ Yes ◯ No
Obs. 8 - Activity Code	◯ A<br>◯ B<br>◯ C<br>◯ D<br>◯ E<br>◯ F<br>◯ G<br>◯ H<br>◯ I<br>◯ J<br>◯ K<br>◯ L<br>◯ M<br>◯ N<br>◯ O<br>◯ P<br>◯ Q<br>◯ R<br>◯ S
Obs. 8 - Start Time	________________
Obs. 8 - End Time	________________
Obs. 8 - Notes	________________
Add Obs. 9	◯ Yes ◯ No

Obs. 9 - Activity Code	◯ A<br>◯ B<br>◯ C<br>◯ D<br>◯ E<br>◯ F<br>◯ G<br>◯ H<br>◯ I<br>◯ J<br>◯ K<br>◯ L<br>◯ M<br>◯ N<br>◯ O<br>◯ P<br>◯ Q<br>◯ R<br>◯ S
Obs. 9 - Start Time	______________________
Obs. 9 - End Time	______________________
Obs. 9 - Notes	______________________
Add Obs. 10	◯ Yes ◯ No
Obs. 10 - Activity Code	◯ A<br>◯ B<br>◯ C<br>◯ D<br>◯ E<br>◯ F<br>◯ G<br>◯ H<br>◯ I<br>◯ J<br>◯ K<br>◯ L<br>◯ M<br>◯ N<br>◯ O<br>◯ P<br>◯ Q<br>◯ R<br>◯ S
Obs. 10 - Start Time	______________________
Obs. 10 - End Time	______________________
Obs. 10 - Notes	______________________

Add Obs. 11	◯ Yes ◯ No
Obs. 11 - Activity Code	◯ A<br>◯ B<br>◯ C<br>◯ D<br>◯ E<br>◯ F<br>◯ G<br>◯ H<br>◯ I<br>◯ J<br>◯ K<br>◯ L<br>◯ M<br>◯ N<br>◯ O<br>◯ P<br>◯ Q<br>◯ R<br>◯ S
Obs. 11 - Start Time	__________________________
Obs. 11 - End Time	__________________________
Obs. 11 - Notes	__________________________
Add Obs. 12	◯ Yes ◯ No
Obs. 12 - Activity Code	◯ A<br>◯ B<br>◯ C<br>◯ D<br>◯ E<br>◯ F<br>◯ G<br>◯ H<br>◯ I<br>◯ J<br>◯ K<br>◯ L<br>◯ M<br>◯ N<br>◯ O<br>◯ P<br>◯ Q<br>◯ R<br>◯ S
Obs. 12 - Start Time	__________________________
Obs. 12 - End Time	__________________________

Obs. 12 - Notes	______________________
Add Obs. 13	◯ Yes ◯ No
Obs. 13 - Activity Code	◯ A<br>◯ B<br>◯ C<br>◯ D<br>◯ E<br>◯ F<br>◯ G<br>◯ H<br>◯ I<br>◯ J<br>◯ K<br>◯ L<br>◯ M<br>◯ N<br>◯ O<br>◯ P<br>◯ Q<br>◯ R<br>◯ S
Obs. 13 - Start Time	______________________
Obs. 13 - End Time	______________________
Obs. 13 - Notes	______________________
Add Obs. 14	◯ Yes ◯ No
Obs. 14 - Activity Code	◯ A<br>◯ B<br>◯ C<br>◯ D<br>◯ E<br>◯ F<br>◯ G<br>◯ H<br>◯ I<br>◯ J<br>◯ K<br>◯ L<br>◯ M<br>◯ N<br>◯ O<br>◯ P<br>◯ Q<br>◯ R<br>◯ S
Obs. 14 - Start Time	______________________

Obs. 14 - End Time	______________________
Obs. 14 - Notes	______________________
Add Obs. 15	◯ Yes ◯ No
Obs. 15 - Activity Code	◯ A<br>◯ B<br>◯ C<br>◯ D<br>◯ E<br>◯ F<br>◯ G<br>◯ H<br>◯ I<br>◯ J<br>◯ K<br>◯ L<br>◯ M<br>◯ N<br>◯ O<br>◯ P<br>◯ Q<br>◯ R<br>◯ S
Obs. 15 - Start Time	______________________
Obs. 15 - End Time	______________________
Obs. 15 - Notes	______________________
Add Obs. 16	◯ Yes ◯ No
Obs. 16 - Activity Code	◯ A<br>◯ B<br>◯ C<br>◯ D<br>◯ E<br>◯ F<br>◯ G<br>◯ H<br>◯ I<br>◯ J<br>◯ K<br>◯ L<br>◯ M<br>◯ N<br>◯ O<br>◯ P<br>◯ Q<br>◯ R<br>◯ S

Obs. 16 - Start Time	__________________________________
Obs. 16 - End Time	__________________________________
Obs. 16 - Notes	__________________________________
Add Obs. 17	◯ Yes ◯ No
Obs. 17 - Activity Code	◯ A<br>◯ B<br>◯ C<br>◯ D<br>◯ E<br>◯ F<br>◯ G<br>◯ H<br>◯ I<br>◯ J<br>◯ K<br>◯ L<br>◯ M<br>◯ N<br>◯ O<br>◯ P<br>◯ Q<br>◯ R<br>◯ S
Obs. 17 - Start Time	__________________________________
Obs. 17 - End Time	__________________________________
Obs. 17 - Notes	__________________________________
Add Obs. 18	◯ Yes ◯ No

Obs. 18 - Activity Code	◯ A<br>◯ B<br>◯ C<br>◯ D<br>◯ E<br>◯ F<br>◯ G<br>◯ H<br>◯ I<br>◯ J<br>◯ K<br>◯ L<br>◯ M<br>◯ N<br>◯ O<br>◯ P<br>◯ Q<br>◯ R<br>◯ S
Obs. 18 - Start Time	__________________________________
Obs. 18 - End Time	__________________________________
Obs. 18 - Notes	__________________________________
Add Obs. 19	◯ Yes ◯ No
Obs. 19 - Activity Code	◯ A<br>◯ B<br>◯ C<br>◯ D<br>◯ E<br>◯ F<br>◯ G<br>◯ H<br>◯ I<br>◯ J<br>◯ K<br>◯ L<br>◯ M<br>◯ N<br>◯ O<br>◯ P<br>◯ Q<br>◯ R<br>◯ S
Obs. 19 - Start Time	__________________________________
Obs. 19 - End Time	__________________________________
Obs. 19 - Notes	__________________________________

Add Obs. 20	◯ Yes ◯ No
Obs. 20 - Activity Code	◯ A<br>◯ B<br>◯ C<br>◯ D<br>◯ E<br>◯ F<br>◯ G<br>◯ H<br>◯ I<br>◯ J<br>◯ K<br>◯ L<br>◯ M<br>◯ N<br>◯ O<br>◯ P<br>◯ Q<br>◯ R<br>◯ S
Obs. 20 - Start Time	__________________________
Obs. 20 - End Time	__________________________
Obs. 20 - Notes	__________________________
Add Obs. 21	◯ Yes ◯ No
Obs. 21 - Activity Code	◯ A<br>◯ B<br>◯ C<br>◯ D<br>◯ E<br>◯ F<br>◯ G<br>◯ H<br>◯ I<br>◯ J<br>◯ K<br>◯ L<br>◯ M<br>◯ N<br>◯ O<br>◯ P<br>◯ Q<br>◯ R<br>◯ S
Obs. 21 - Start Time	__________________________
Obs. 21 - End Time	__________________________

Obs. 21 - Notes

____

Add Obs. 22 ◯ Yes ◯ No

Obs. 22 - Activity Code
◯ A
◯ B
◯ C
◯ D
◯ E
◯ F
◯ G
◯ H
◯ I
◯ J
◯ K
◯ L
◯ M
◯ N
◯ O
◯ P
◯ Q
◯ R
◯ S

Obs. 22 - Start Time

____

Obs. 22 - End Time

____

Obs. 22 - Notes

____

Add Obs. 23 ◯ Yes ◯ No

Obs. 23 - Activity Code
◯ A
◯ B
◯ C
◯ D
◯ E
◯ F
◯ G
◯ H
◯ I
◯ J
◯ K
◯ L
◯ M
◯ N
◯ O
◯ P
◯ Q
◯ R
◯ S

Obs. 23 - Start Time

____

Obs. 23 - End Time	__________________________________
Obs. 23 - Notes	__________________________________
Add Obs. 24	◯ Yes ◯ No
Obs. 24 - Activity Code	◯ A<br>◯ B<br>◯ C<br>◯ D<br>◯ E<br>◯ F<br>◯ G<br>◯ H<br>◯ I<br>◯ J<br>◯ K<br>◯ L<br>◯ M<br>◯ N<br>◯ O<br>◯ P<br>◯ Q<br>◯ R<br>◯ S
Obs. 24 - Start Time	__________________________________
Obs. 24 - End Time	__________________________________
Obs. 24 - Notes	__________________________________
Add Obs. 25	◯ Yes ◯ No
Obs. 25 - Activity Code	◯ A<br>◯ B<br>◯ C<br>◯ D<br>◯ E<br>◯ F<br>◯ G<br>◯ H<br>◯ I<br>◯ J<br>◯ K<br>◯ L<br>◯ M<br>◯ N<br>◯ O<br>◯ P<br>◯ Q<br>◯ R<br>◯ S

Obs. 25 - Start Time	__________________
Obs. 25 - End Time	__________________
Obs. 25 - Notes	__________________
Add Obs. 26	◯ Yes ◯ No
Obs. 26 - Activity Code	◯ A<br>◯ B<br>◯ C<br>◯ D<br>◯ E<br>◯ F<br>◯ G<br>◯ H<br>◯ I<br>◯ J<br>◯ K<br>◯ L<br>◯ M<br>◯ N<br>◯ O<br>◯ P<br>◯ Q<br>◯ R<br>◯ S
Obs. 26 - Start Time	__________________
Obs. 26 - End Time	__________________
Obs. 26 - Notes	__________________
Add Obs. 27	◯ Yes ◯ No

Obs. 27 - Activity Code	◯ A<br>◯ B<br>◯ C<br>◯ D<br>◯ E<br>◯ F<br>◯ G<br>◯ H<br>◯ I<br>◯ J<br>◯ K<br>◯ L<br>◯ M<br>◯ N<br>◯ O<br>◯ P<br>◯ Q<br>◯ R<br>◯ S
Obs. 27 - Start Time	__________________________________
Obs. 27 - End Time	__________________________________
Obs. 27 - Notes	__________________________________
Add Obs. 28	◯ Yes ◯ No
Obs. 28 - Activity Code	◯ A<br>◯ B<br>◯ C<br>◯ D<br>◯ E<br>◯ F<br>◯ G<br>◯ H<br>◯ I<br>◯ J<br>◯ K<br>◯ L<br>◯ M<br>◯ N<br>◯ O<br>◯ P<br>◯ Q<br>◯ R<br>◯ S
Obs. 28 - Start Time	__________________________________
Obs. 28 - End Time	__________________________________
Obs. 28 - Notes	__________________________________

Add Obs. 29	◯ Yes ◯ No
Obs. 29 - Activity Code	◯ A<br>◯ B<br>◯ C<br>◯ D<br>◯ E<br>◯ F<br>◯ G<br>◯ H<br>◯ I<br>◯ J<br>◯ K<br>◯ L<br>◯ M<br>◯ N<br>◯ O<br>◯ P<br>◯ Q<br>◯ R<br>◯ S
Obs. 29 - Start Time	__________________________________
Obs. 29 - End Time	__________________________________
Obs. 29 - Notes	__________________________________
Add Obs. 30	◯ Yes ◯ No
Obs. 30 - Activity Code	◯ A<br>◯ B<br>◯ C<br>◯ D<br>◯ E<br>◯ F<br>◯ G<br>◯ H<br>◯ I<br>◯ J<br>◯ K<br>◯ L<br>◯ M<br>◯ N<br>◯ O<br>◯ P<br>◯ Q<br>◯ R<br>◯ S
Obs. 30 - Start Time	__________________________________
Obs. 30 - End Time	__________________________________

Obs. 30 - Notes

____

Comments:

____

Audit Trail

Name of Data Entry Person

____

Date of Data Entry

____
(Kindly change the date every time a change is made to the data)

Livestock Form LS

LS01. What happens to your household garbage?	◯ Municipal waste<br>◯ Burnt<br>◯ In the yard<br>◯ Dump<br>◯ Other
Specify Other	__________________________
LS02. Does the household own, keep or look after any animals (cats, dogs, cattle, chickens, ducks etc.)?	◯ Yes<br>◯ No
LS03. Do your animals have access to the household garbage?	◯ Yes<br>◯ No
LS04. What domestic animal/s does the household own, keep or look after?	☐ Cattle<br>☐ Fowl (eg. Chickens, geese, duck)<br>☐ Pig<br>☐ Sheep<br>☐ Goat<br>☐ Horse<br>☐ Donkey<br>☐ Cat<br>☐ Dog<br>☐ Pigeon<br>☐ Other
Cattle Count	__________________________
Fowl Count	__________________________
Pig Count	__________________________
Sheep Count	__________________________
Goat Count	__________________________
Horse Count	__________________________
Donkey Count	__________________________
Cat Count	__________________________
Dog Count	__________________________

Pigeon Count

____

Specify Other

____

Other Count

____

LS05. Do any animals live or stay overnight inside the house where other people sleep?

- ◯ Yes
- ◯ No

LS05. Tick all the animals that sleep in the house

- ☐ Cattle
- ☐ Fowl (eg. Chickens, geese, duck)
- ☐ Pig
- ☐ Sheep
- ☐ Goat
- ☐ Horse
- ☐ Donkey
- ☐ Cat
- ☐ Dog
- ☐ Pigeon
- ☐ Other

Specify Other

____

LS06. Do any of the animals live or stay overnight in the yard or kraal around the household?

- ◯ Yes
- ◯ No

LS06. Tick all those that sleep in the yard or kraal around the household

- ☐ Cattle
- ☐ Fowl (eg. Chickens, geese, duck)
- ☐ Pig
- ☐ Sheep
- ☐ Goat
- ☐ Horse
- ☐ Donkey
- ☐ Cat
- ☐ Dog
- ☐ Pigeon
- ☐ Other

Specify Other

____

LS07. Do any of the animals sleep in a field or kraal far from the household?

- ◯ Yes
- ◯ No

LS07. Tick all the animals that sleep in a field far from the household	☐ Cattle<br>☐ Fowl (eg. Chickens, geese, duck)<br>☐ Pig<br>☐ Sheep<br>☐ Goat<br>☐ Horse<br>☐ Donkey<br>☐ Cat<br>☐ Dog<br>☐ Pigeon<br>☐ Other
Specify Other	__________________________________
L08. How far is the closest kraal from where people sleep and/or eat?	◯ 1-10 metres<br>◯ 11-50 metres<br>◯ 51-100 metres<br>◯ 101-500 metres<br>◯ .501-1km<br>◯ +1km<br>◯ Not Applicable

Only answer this section for those households that report owning at least one of the following animals: poultry, cattle, sheep, pigs, goat

LS09. Who looks after the animal/s	☐ Animals looked after by someone who is not a household member
LS09. Who looks after the animal/s 1)	__________________________________
LS09. Who looks after the animal/s 2)	__________________________________
LS09. Who looks after the animal/s 3)	__________________________________
LS09. Who looks after the animal/s 4)	__________________________________
LS10. In the last 6 months have any of your animals been slaughtered/killed?	◯ Yes<br>◯ No
LS11. Tick all the animals that were slaughtered/killed in the and indicate how many of each animal were slaughtered/killed	☐ Cattle<br>☐ Sheep<br>☐ Pig<br>☐ Goat<br>☐ Fowls (eg. Chickens, geese, ducks)<br>☐ Pigeon
Cattle slaughtered/killed	__________________________________
Sheep slaughtered/killed	__________________________________

Pig slaughtered/killed	______________________
Goat slaughtered/killed	______________________
Fowls slaughtered/killed	______________________
Pigeon slaughtered/killed	______________________
LS12. In the last 6 months, were any of the animals slaughtered/killed at the household?	◯ Yes<br>◯ No
List the household members that slaughtered and/or assisted in slaughtering the animals	☐ Animals slaughtered by someone who is not a household member
LS13. List the household members that slaughtered and/or assisted in slaughtering the animals<br>1)	______________________
LS13. List the household members that slaughtered and/or assisted in slaughtering the animals<br>2)	______________________
LS13. List the household members that slaughtered and/or assisted in slaughtering the animals<br>3)	______________________
LS13. List the household members that slaughtered and/or assisted in slaughtering the animals<br>4)	______________________
LS13. List the household members that slaughtered and/or assisted in slaughtering the animals<br>5)	______________________
LS14. Tick all the animals that were slaughtered at the household in the last 6 months	☐ Cattle<br>☐ Sheep<br>☐ Pig<br>☐ Goat<br>☐ Fowls (eg. Chickens, geese, ducs)<br>☐ Pigeon<br>☐ Not Applicable
LS15. Were any of the animals taken to a slaughter house?	☐ Cattle<br>☐ Sheep<br>☐ Pig<br>☐ Goat<br>☐ Fowls (eg. Chickens, geese, ducs)<br>☐ Pigeon<br>☐ Not Applicable

LS16. What happened with the slaughtered/killed animal?	◯ It was eaten by the household members<br>◯ It was sold for money<br>◯ It was thrown away/disposed of<br>◯ It was shared with the community<br>◯ Ritual purposes<br>◯ Other
Specify Other	__________________________
LS17. Do you and members in your household drink milk that comes from your cows/goat/sheep?	◯ Yes<br>◯ No
LS18. If 'Yes', how do you drink the milk?	◯ Raw milk - no heating or preparation<br>◯ Boil milk for a short time (a few minutes)<br>◯ Boil milk for a long time (around half an hour)<br>◯ Other
Specify Other	__________________________
LS19. Do you know if any of your animals have been tested for Tuberculosis in the past 12 months?	◯ Yes<br>◯ No
LS20. Tick all the animals that were tested for TB in the last 12 months	☐ Cattle<br>☐ Pig<br>☐ Sheep<br>☐ Goat<br>☐ Horse<br>☐ Donkey<br>☐ Cat<br>☐ Dog<br>☐ Other
Specify Other	__________________________

Animals with Sickness and Disease

LS21. In the last 6 months have any of your animals had to go to the vet for sickness or disease?	◯ Yes<br>◯ No
LS22. Have any of your animals and any of these symptoms:	☐
LS23. In the last 6 months have any of your animals died because of sickness or disease?	◯ Yes<br>◯ No

LS24. Tick all the animals that in last 6 months died because of sickness or disease and indicate how many of each animal died	☐ Cattle<br>☐ Fowls (eg. Chickens, geese, ducks)<br>☐ Pig<br>☐ Sheep<br>☐ Goat<br>☐ Horse<br>☐ Donkey<br>☐ Cat<br>☐ Dog<br>☐ Pigeon<br>☐ Other
Specify Other	______________________
Cattle Count	______________________
Fowl Count	______________________
Pig Count	______________________
Sheep Count	______________________
Goat Count	______________________
Horse Count	______________________
Donkey Count	______________________
Cat Count	______________________
Dog Count	______________________
Pigeon Count	______________________
[ls24_other] Count	______________________
LS24. What happened to the meat of the animal that died?	◯ It was eaten<br>◯ It was sold<br>◯ It was given to people<br>◯ It was thrown out<br>◯ Other
Specify Other	______________________

LS25. Would you consider having a small skin test done on all of your animals?

◯ Yes
◯ No

LS26. If 'No', why not?

____

LS27. On average, how much would you sell each of your animals to us if we wanted to buy it?

☐ Cattle
☐ Fowls (eg. Chickens, geese, ducks)
☐ Pig
☐ Sheep
☐ Goat
☐ Horse
☐ Donkey
☐ Cat
☐ Dog
☐ Pigeon
☐ Other

Specify Other

____

Cattle Selling Price

____
(R (ZAR))

Fowl Selling Price

____
(R (ZAR))

Pig Selling Price

____
(R (ZAR))

Sheep Selling Price

____
(R (ZAR))

Goat Selling Price

____
(R (ZAR))

Horse Selling Price

____
(R (ZAR))

Donkey Selling Price

____
(R (ZAR))

Cat Selling Price

____
(R (ZAR))

Dog Selling Price

____
(R (ZAR))

Pigeon Selling Price	______________ (R (ZAR))
[ls27_other] Selling Price	______________ (R (ZAR))

Only answer this section for those households that do NOT own any animals

LS28. Do you and members in your household ever drink raw (unpasteurized, un-boiled milk)?	◯ Yes<br>◯ No
LS29: If yes, how often	◯ Always<br>◯ Very Often<br>◯ Sometimes<br>◯ Rarely
LS30. Do you and members in your household ever buy meat from an informal butcher in the area?	◯ Yes<br>◯ No
LS31: If yes, ask how often	◯ Always<br>◯ Very Often<br>◯ Sometimes<br>◯ Rarely
LS32. Do any of the household members look after other people's/neighbours/community members' animals (example Herding cattle)?	◯ Yes<br>◯ No
LS33. In the last 6 months, has anyone in the household attended a function (funeral, wedding) where an animal was slaughtered and the meat was eaten?	◯ Yes<br>◯ No
How many functions	______________
LS34. In the last 6 months, have your neighbours/community members slaughtered any animals?	◯ Yes<br>◯ No
LS35. In the last 6 months, have you eaten any meat that you got/were given from your neighbours/community members after they slaughtered the animal?	◯ Yes<br>◯ No
LS36: Date form completed	______________
LS37: Name of Person completing this form	______________

Audit Trail

Name of Data Entry Person

____

Date of Data Entry

____
(Kindly change the date every time a change is made to the data)
